# Effects of a 12-week intrinsic foot muscle strengthening training (STIFF) on gait in older adults: a parallel randomized controlled trial protocol

**DOI:** 10.1186/s13102-024-00944-z

**Published:** 2024-07-20

**Authors:** Lydia Willemse, Eveline J. M. Wouters, Martijn F. Pisters, Benedicte Vanwanseele

**Affiliations:** 1 Fontys University of Applied Sciences, PO Box 347, Eindhoven, AH 5600 The Netherlands; 2 Department of Movement Sciences, KU Leuven, Tervuursevest 101 - box 1500, Louvain, 3001 Belgium; 3 Tranzo, School of Social and Behavioral Sciences, Tilburg University, PO Box 90153, Tilburg, LE 5000 The Netherlands; 4 Department of Rehabilitation, Physiotherapy Science & Sport, UMC Utrecht Brain Center, Utrecht University, PO Box 85500, Utrecht, GA 3508 The Netherlands; 5 Center for Physical Therapy Research and Innovation in Primary Care, Julius Health Care Centers, PO Box 85500, Utrecht, GA 3508 The Netherlands

**Keywords:** Foot muscles, Falling, Gait, Older adults, Exercise

## Abstract

**Background:**

Falling is highly prevalent among older adults and has serious impact. Age-induced mobility impairments, such as gait modifications, are strongly associated with increased fall risk. Among fall prevention interventions, those including exercises are most effective. However, there is an urgent need to further improve these kinds of interventions. Strengthening the plantar intrinsic foot muscles might benefit mobility in older adults, which may contribute to the reduction of fall risk. The aim of this paper is to provide a protocol to investigate the effect of a plantar intrinsic foot muscle strengthening training versus no training on gait and intrinsic foot muscle function in older adults who are involved in a functional exercise program.

**Methods:**

For this assessor-blinded RCT, older adults (> 65 years) are recruited who are involved in a group-based functional exercise program. Eligibility criteria include: being able to ambulate 10 m barefoot without using a walking aid and reporting to have either fear of falling or experienced a fall in the previous 12 months or have difficulties with mobility, gait, or balance in daily life. Participants are randomly assigned to an intervention and a control group. The intervention group follows a 12-week plantar intrinsic foot muscle strengthening training. The training consists of isolated and functional foot exercises to be performed 5 times a week, each session lasting approximately 20 min. The training is supervised once a week and the intensity gradually increases based on the participant’s progression. Both groups keep a diary to report physical activities, fall incidents and movement related discomfort. The control condition is limited to keeping this diary. Data are collected at baseline and post-intervention. The trial outcomes are the between group differences in the mean change from baseline in maximum gait speed (primary outcome measure), capacity and strength of the plantar intrinsic foot muscles, foot and ankle biomechanics during gait, and various other fall risk-related variables. ANCOVA’s are used to analyze the trial outcomes.

**Discussion:**

The results of this RCT will offer recommendations, related to plantar intrinsic foot muscle strengthening, to existing fall preventive exercise programs.

**Trial registration:**

The trial is registered in the United States National Library of Medicine through ClinicalTrials.gov (NCT05531136, 07/26/2022).

**Supplementary Information:**

The online version contains supplementary material available at 10.1186/s13102-024-00944-z.

## Background

Falling is highly prevalent among older adults and has serious impact. More than one third of the adults aged above 65 years fall at least once a year [1]. Once an individual has experienced a fall, well-being is often compromised as a result of injuries or by fearing another fall incident [2, 3]. Increased fall risk is strongly associated with age-induced mobility impairments, such as gait modifications and balance deficits [2, 4, 5]. Among fall prevention interventions, those involving a functional exercise program aimed at improving mobility (i.e., gait, balance, coordination and functional task training [6]) seem to be most effective, reducing the rate of falls by 24% [7]. To further reduce this rate, there is an urgent need for strategies to improve these fall prevention interventions and, more generally, ongoing functional exercise programs for older adults [7].

Functional exercise programs, including fall preventive exercise interventions, are established without noticeable understanding of the plantar intrinsic foot muscles (PIFMs), while there are indications that these muscles have a role in fall related aspects of mobility. The PIFMs stabilize and stiffen the foot [8, 9] and consequently contribute to balance and propulsive gait [10, 11]. These mobility aspects are reflected in maximum gait speed, which has been associated with falling [12, 13] and toe flexor strength [14]. Weakness of the PIFMs in older adults [15] may thus have a detrimental effect on mobility and fall risk. Indeed, it was found that toe flexor weakness predicts falling in older adults [16, 17]. Some evidence exists for the beneficial effect of PIFM strengthening on propulsive capacity during gait, even in a population with unaffected PIFMs and unimpaired mobility [18]. This suggests that strengthening the PIFMs might improve mobility in older adults, which may contribute to the reduction of fall risk. Yet, this needs to be examined.

Several foot and ankle exercise interventions that also target the PIFMS have been investigated in older adults [19–23]. While beneficial results were shown in separate studies for toe flexor strength [20, 21] and gait parameters [23], the PIFMs were not examined concurrently with mobility outcomes. Consequently, it remains unclear how improvements in mobility are linked to possibly enhanced function of the PIFMs. Now that there is growing evidence for the importance of the PIFMs in relation to mobility, a high-quality study that addresses this gap is needed to evaluate the effect of training the PIFMs in older adults. The outcome will enable adequate advice towards fall preventive exercise interventions with regard to the incorporation of PIFMs’ exercises. Therefore, we set up a randomized controlled trial (RCT) that aims to examine the effect of a PIFM strengthening training on fall-related mobility parameters in older adults.

The primary hypothesis in this study is that a PIFM strengthening training versus no PIFM strengthening training increases maximum gait speed in older adults who are involved in a functional exercise program. To investigate the trainability of the PIFMs and how this translates into improved mobility in this specific population, this study further hypothesizes that this PIFM strengthening training has a beneficial effect on PIFM capacity, isometric toe flexor strength, foot and ankle biomechanics during gait, comfortable gait speed, step length, balance during gait, self-reported judgement of mobility, physical activity, fall incidents, fear of falling and physical functioning.

## Methods

### Design

The study design is an assessor-blinded superiority RCT with two parallel groups. Participants, older adults who are involved in a functional exercise program at the time of recruitment, are randomly assigned with a 1:1 ratio to the PIFM strengthening training group and a control group. The functional exercise program is delivered outside the scope of this study and is continued by the participants as usual. The PIFM strengthening training is delivered as a separate program for the purpose of this study. Measurements take place at baseline and directly after the 12-week intervention period at the movement analysis laboratory at Fontys University of Applied Sciences, Eindhoven, The Netherlands. The trial is registered in the United States National Library of Medicine through ClinicalTrials.gov (NCT05531136). Table 1 shows the key registration data. The protocol is approved by the medical research ethics committee of Maxima Medical Center, Veldhoven, The Netherlands (CCMO nr. NL80110.015.21). The study protocol is reported according to the SPIRIT (Standard Protocol Items: Recommendations for Interventional Trials) statement 2013 [24] (see Additional file 1 for the completed SPIRIT checklist) and the CONSORT (Consolidated Standards of Reporting Trials) Statement for Randomized Trials of Nonpharmacologic Treatments [25] (see Additional file 2 for the completed CONSORT checklist).

**Table 1:** Trial registration data

Primary Registry and Trial Identifying Number	*ClinicalTrials.gov: NCT05531136*
Date of Registration in Primary Registry	*07/26/2022*
Secondary Identifying Numbers	*CCMO: NL80110.015.21* *NWO: 023.013.063*
Source(s) of Monetary or Material Support	*Funding agency: The Dutch Research Council (NWO)*
Primary Sponsor	*Fontys University of Applied Sciences, Eindhoven, The Netherlands*
Secondary Sponsor(s)	*n/a*
Contact for Public Queries	*Lydia Willemse, MSc.: lydia.willemse@fontys.nl;* + *31885089836; PO Box 347, 5600 AH Eindhoven, The Netherlands*
Contact for Scientific Queries	*Lydia Willemse, MSc.: lydia.willemse@fontys.nl;* + *31885089836; PO Box 347, 5600 AH Eindhoven, The Netherlands*
Public Title	*Effect of a Foot Muscle Strengthening Program in Mobile Older Adults (STIFF3)*
Scientific Title	*Effect of a Foot Muscle Strengthening Program in Mobile Older Adults (STIFF3)*
Countries of Recruitment	*The Netherlands*
Health Condition(s) or Problem(s) Studied	*Fall risk*
Intervention(s)	*Arm Title 1: Foot strengthening training, Arm Type 1: Experimental, Arm Description 1: 12-week foot strengthening training in addition to an already joined functional exercise program. The training consists of foot strengthening exercises prescribed for 5 daily sessions a week, of which 1 supervised, 20 min per session on top of the regular exercise program to prevent falling. The foot strengthening training is progressing and consists of isolated and functional exercises. Participants keep a training diary.* *Arm Title 2: Control, Arm Type 2: No Intervention, Arm Description 2: The control group continues the functional exercise program as usual. The subjects in this group are asked to keep a diary in which the subjects weekly report other physical activities, fall incidents and mobility related discomfort. The trainer calls the participants in the control group every week to pay attention to these topics.*
Key Inclusion and Exclusion Criteria	*Minimum Age: 65 Years; Sex: All; Accepts Healthy Volunteers: Yes; Criteria: Inclusion Criteria: be 65 years of age or over, be able to ambulate 10 m barefoot without using a walking aid, engage in a functional exercise program delivered to a group of older adults by an educated or certified physical therapist or trainer (e.g., fall preventive exercise program, senior fit programs), report to have 1) fear of falling OR 2) experienced a fall in the previous 12 months OR 3) difficulties with mobility, gait, or balance, be able to arrange their own transport to the movement analysis laboratory. Exclusion Criteria: The respondent is a mentally incapacitated adult, Self-reported presence of any disorder interfering with the execution of the exercise program.*
Study Type	*Study Type: Interventional; Primary Purpose: Prevention; Study Phase: n/a; Interventional Study Model: Parallel; Model Description: The study design is an assessor-blinded RCT with two parallel groups. Participants are randomly assigned in a 1:1 ratio to either the intervention or the control group by the use of a computer-generated randomization list managed by an independent project administrator. Blocking, the size of the blocks undisclosed, is applied in order to ensure the balanced allocation at several time points in the trial; Number of Arms: 2; Masking: Investigator, Outcomes Assessor; Masking Description: Once a participant has accomplished the baseline measurements, the assessor requests group allocation. The project administrator then sends the allocation to the trainer, who assigns the participant to the allocated group. The nature of the intervention precludes blinding of the participants and the trainers, however staff members involved in the recruitment of participants and in the assessment of measurement variables remain blinded to the group allocation until the post-processing of data that involves any subjectivity has been completed. Allocation: Randomized.*
Date of First Enrollment	*08/11/2022*
Sample Size	*42 [anticipated]*
Recruitment Status	*Recruiting*
Primary Outcome(s)	*Title: maximum gait speed; Description: The post-intervention difference between the intervention and control group in maximum gait speed, Time Frame: 12 weeks.*
Key Secondary Outcomes	*Title: Foot muscles’ morphology derived from ultrasound imaging, Time Frame: 12 weeks;* *Title: Lower extremity biomechanics during gait assessed with 3D motion and ground reaction force capturing, Time Frame: 12 weeks; Title: Spatiotemporal gait parameters assessed with 3D motion and ground reaction force, Time Frame: 12 weeks; Title: Balance during gait assessed with 3D motion and ground reaction force capturing, Time Frame: 12 weeks; Title: Self-reported mobility limitations, Time Frame: 12 weeks; Title: Physical activity engagement, Time Frame: 12 weeks; Title: Fall incidents during the intervention, Time Frame: 12 weeks; Title: Fear of falling assessed by the (Falls Efficacy Scale-International) FES-I questionnaire, Time Frame: 12 weeks; Title: Isometric toe flexor strength assessed by a pressure plate during maximal toe press, Time Frame: 12 weeks; Title: Physical functioning assessed by the Short Physical Performance Battery (SPPB), Time Frame: 12 weeks;*
Ethics Review	*Board Status: Submitted, approved; Approval Number: W21.104; Board Name: METC; Board Affiliation: Maxima MC; Board Contact: Phone:* + *31,408,889,528; Email: metc@mmc.nl; Address: De Run 4600, Veldhoven, The Netherlands*
Completion date	*n/a*
Summary Results	*n/a*
IPD sharing statement	*n/a*

### Participants

#### Recruitment

**Fig. 1:**
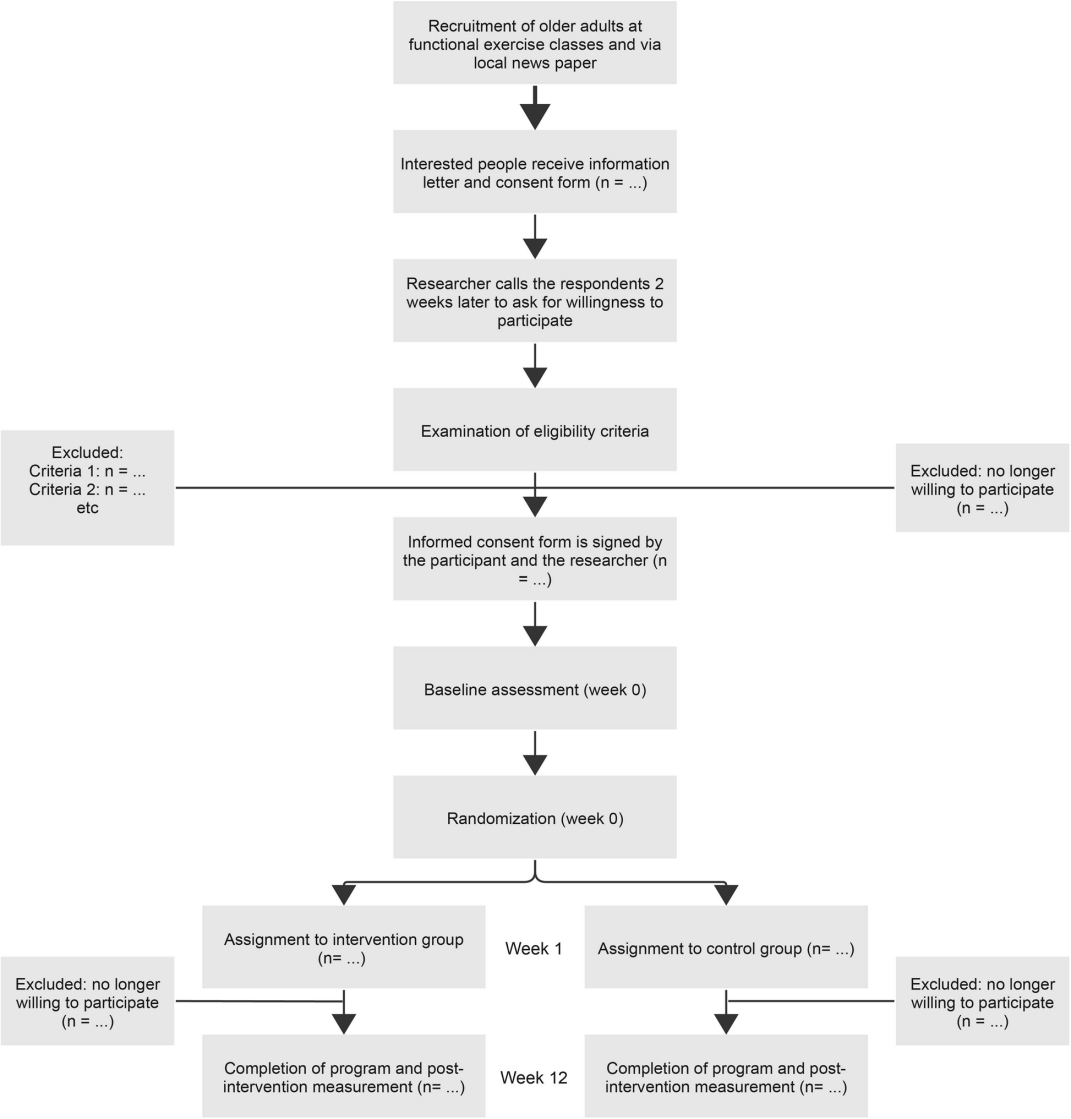
Flow of participants

**Fig. 2:**
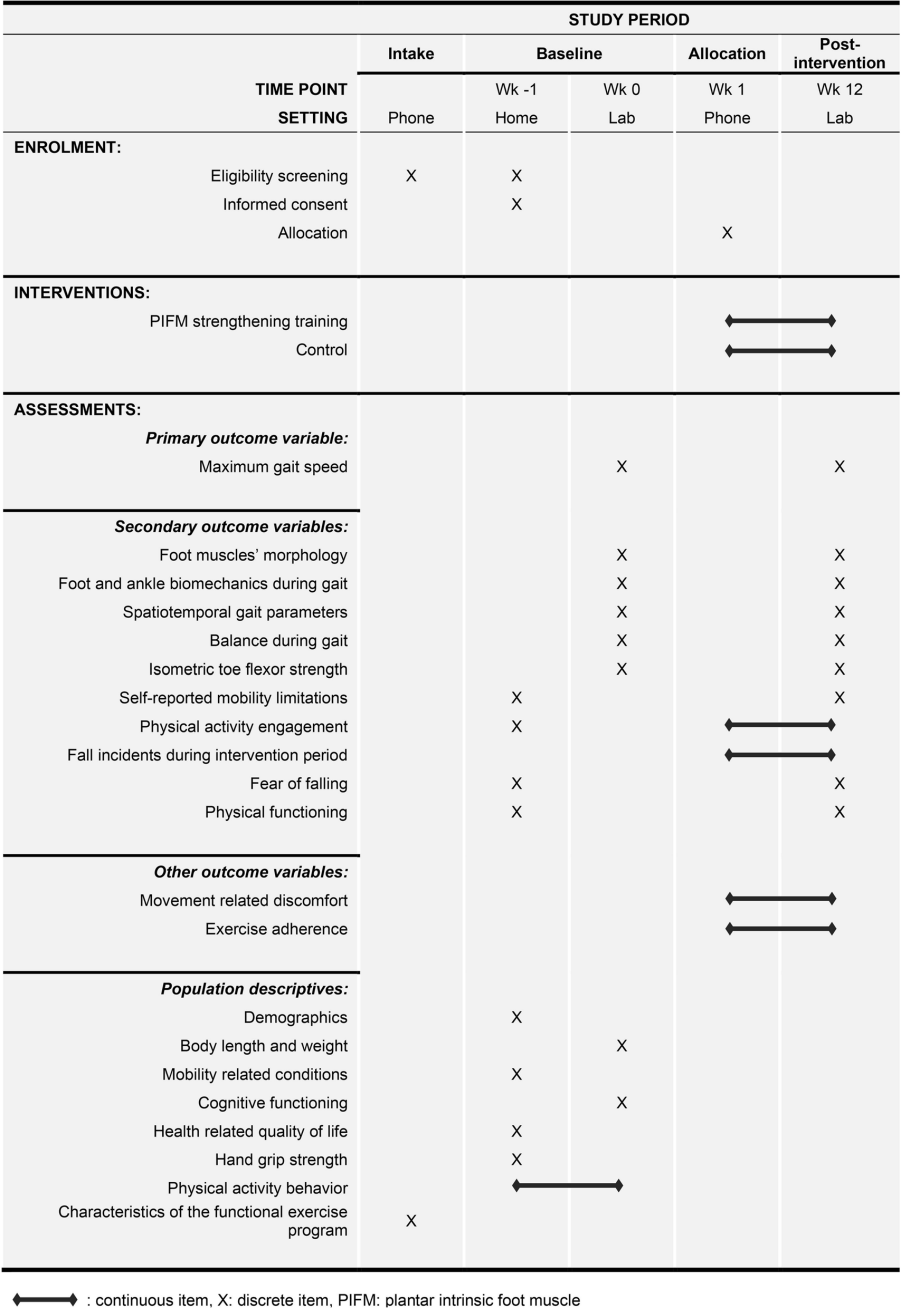
Participant timeline showing enrolment, interventions, and assessments

Older adults (> 65 years) who are involved in a group-based functional exercise program are recruited at fall prevention classes and senior sports and exercise classes in and around the city of Eindhoven, The Netherlands. Participants are recruited by the primary investigator (LW) via verbal communication about the study outline to the group of older adults and via posters and leaflets at the site of the exercise classes, and in a local newspaper. Interested people are asked to share their contact details and they receive a hard copy of the information letter and the consent form. After two weeks, the researcher calls the respondent, providing the opportunity to ask questions and to ask for willingness to participate in the study. If so, the eligibility is examined based on the selection criteria. See Fig. 1 for the flow of participants and Fig. 2 for the participant timeline.

#### Selection

In order to be eligible to participate in the study, respondents should 1) be 65 years of age or over, 2) be able to ambulate 10 m barefoot without using a walking aid, 3) be involved in a functional exercise program delivered to a group of older adults by an educated or certified physical therapist or instructor (e.g., fall preventive exercise program, senior fit programs), 4) report to have fear of falling or to have experienced a fall in the previous 12 months or to have difficulties with mobility, gait, or balance in daily life, 5) be able to arrange their own transport to the movement analysis laboratory. Respondents who report presence of any disorder interfering with adherence or the execution of the exercises are excluded. To this end, a general explanation of the intervention and an exemplary exercise (i.e., toe pressing) is given. Mentally incapacitated individuals are also excluded from participation. The informed consent form is signed before data collection, first by the participant and then by the researcher. After having received written informed consent to participate, the participant is included in the study.

#### Sample size

We test the primary hypothesis that the mean change from baseline at post-intervention in maximum gait speed is in the positive direction and larger in the intervention group than in the control group. Using the anchor-based approach [26] for the difference in maximum gait speed between older adults at high (M: 1.54 m/s, sd: 0.37 m/s) and low (M: 1.83 m/s, sd: 0.33 m/s) fall risk [13] and between older adults with (M: 0.96 m/s, sd: 0.32 m/s) and without (M: 1.23 m/s, sd: 0.36 m/s) a fall history [12], we decided the minimal clinically important effect size to be $d=0.79$ according to these formulae:

$$d= \frac{1.83-1.54}{\sqrt{\frac{{0.33}^{2}+{0.37}^{2}}{2}}}=0.83$$

$$d= \frac{1.23-0.96}{\sqrt{\frac{{0.36}^{2}+{0.32}^{2}}{2}}}=0.79$$

For this effect size, which equals *η*^*2*^ = *0.135* [27]*,* to be detected with ANCOVA while applying $\beta =0.8$ and $\alpha =0.05$ (one-tailed), the sample size should be $n=42$ according to our calculation in G*power 3.1.9.2 software.

### Randomization and blinding

Participants are randomly assigned in a 1:1 ratio to either the intervention or the control group by the use of a computer-generated (randomizer.org) randomization list managed by an independent project administrator. Blocked randomization, the size of the blocks being undisclosed, is applied to ensure the balanced allocation at several time points during the trial. Once a participant has accomplished the baseline measurements, the primary investigator asks the project administrator to send the group allocation to the trainer, who assigns the participant to the allocated group. The nature of the intervention precludes blinding of the participants and the trainers, however the primary investigator and assistant assessors remain blinded to the group allocation until the post-processing of data that is exposed to subjectivity (i.e., segmentation of ultrasound images) is completed. At the end of the post-intervention measurement, the group assignment is guessed by the primary investigator to evaluate the success of the blinding procedures. The primary investigator is the same person who performs the data analysis.

### Interventions

#### PIFM training program

**Fig. 3:**
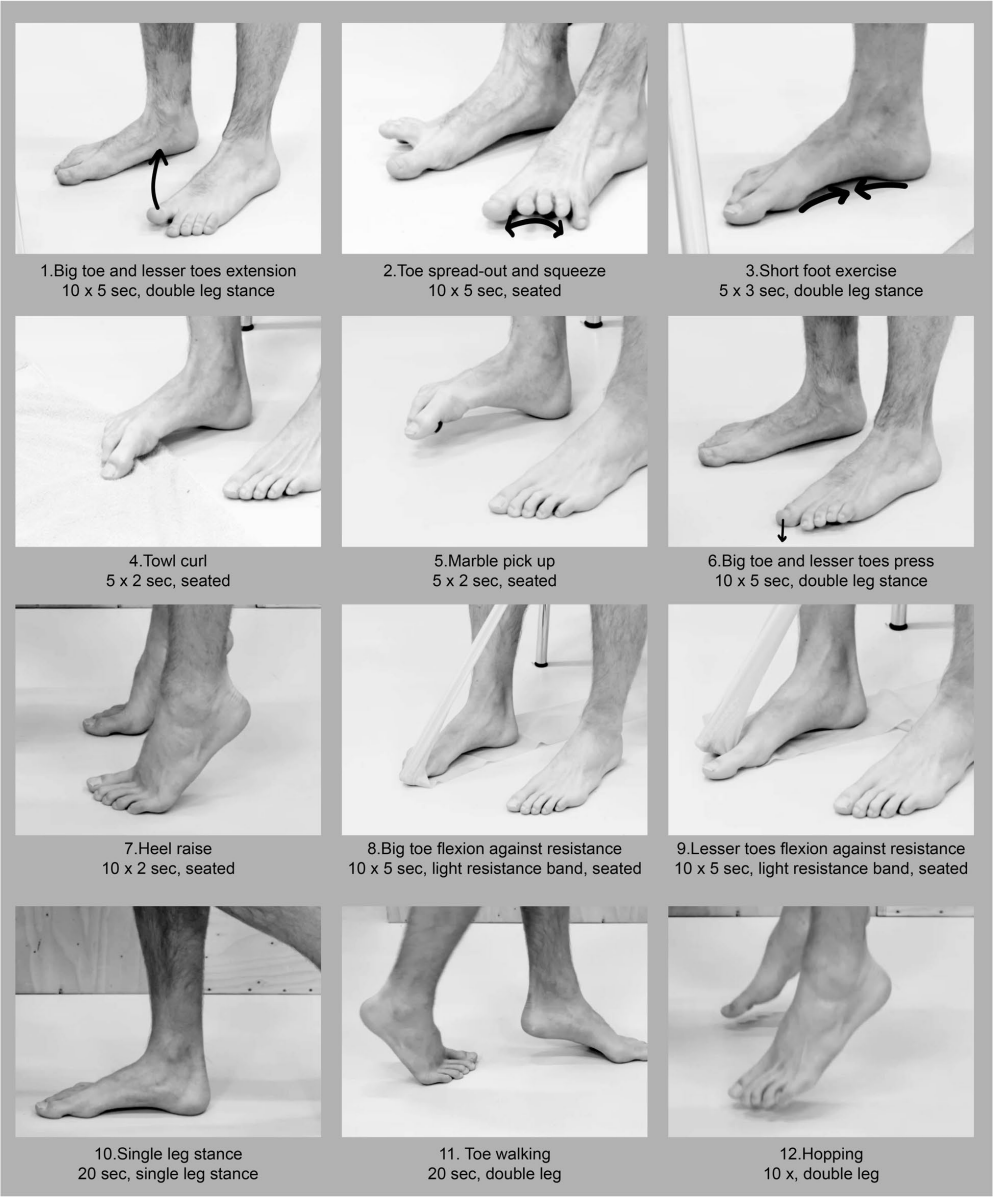
The 12 exercises included in the PIFM strengthening training. For each exercise, the number of repetitions, the contraction/exercise duration and the pose is presented for the easiest intensity level of the training program

The intervention group follows a 12-week PIFM strengthening training. The training has been developed with a design thinking approach [28]. A first draft of the program was based on existing literature concerning foot strengthening programs [20, 29–34], training principles [35] and the behavior change wheel [36]. It then went through several iteration rounds with older adults, (foot and ankle) physiotherapists, podiatrists and a human movement scientist. The final training program prescribes 20 min of PIFM strengthening exercises (see the training guide in Additional file 3). The training consists of both isolated and functional foot exercises, of which the intensity gradually increases based on the participant’s progression, to be executed 5 days a week. Figure 3 shows the exercises included in the training, together with the number of repetitions, the contraction time and pose for the easiest intensity level of the training. Once a week, the training is supervised by a 4th years physiotherapy student who receives extensive education in delivering the training in a standardized fashion prior to delivering the training. A trainer’s guide (see Additional file 4) provides the trainer guidance in delivering the training. A weekly meeting with the trainers and the researcher intends to promote adherence of the trainers to the protocol. In addition to the education of the trainers, the standardization of the training is also achieved by instructional videos of each exercise along with written instructions provided in the training guide (see Additional file 3). This training guide further includes a training log, in which the participant reports the perceived difficulty for each exercise and each training session using a 5-point Likert scale [29]. In addition, the training guide comes along with a diary that serves to monitor adherence to the prescribed program, physical activities, fall incidents and movement related discomfort. For safety reasons, the participant is instructed to report movement related discomfort in the lower extremities immediately to the trainer. In this case, the training will be continued with lesser intensity until the discomfort has disappeared. The participant decides if the supervised training is a group session at Fontys Allied Health Professions or on individual basis delivered at home. Each session commences with a warm-up and ends with a cool-down, including stretching.

At the onset of the training, each participant starts the exercises at the easiest level. When the participant perceives the exercise without any difficulty for 5 consecutive training sessions and the trainer scores maximum motor performance using a 3-point scale, modified from Fraser and Hertel [29], the trainer sets the level of intensity to the next level (see page 6 of the trainer’s guide in Additional file 4). If the duration of the training session exceeds 20 min, for example due to more advanced intensity levels (i.e., more repetitions), the trainer discusses with the participant how to limit the duration of training sessions in accordance with preset restrictions (see page 7 of the trainer’s guide in Additional file 4).

To promote adherence, the training guide visualizes completed sessions, which is discussed with the trainer as part of every supervised session. The personal guidance by the trainer and the participant’s choice for either a home visit by the trainer or joining a group session also anticipates maximum adherence to the exercise regime.

#### Control group

The participants in the control group are asked to keep a diary (see Diary (control) in Additional file 5) in which the participants weekly report other physical activities, fall incidents and movement related discomfort. The trainer calls the participants in the control group every week paying attention to these topics.

### Data collection procedures

Baseline data are collected prior to the group allocation. The outcome variables per time point are presented in the participant timeline, Fig. 2. The baseline data are collected during a home visit and a laboratory session, separated by approximately one week. We introduced the home visit (~ 1 h) as it facilitates a personal introduction to the study and it reduces the duration of the baseline laboratory session, limiting the risk for fatigue. The home visit is completed by trained assistant assessors. The primary investigator is in charge of the data collection at the motion analysis laboratory (baseline and post-intervention; ~ 3 h each) while assisted by assistant assessors. Unilateral outcome variables are taken from the dominant stance leg, determined by single leg stance. The trainer instructs the participant right before the post-intervention measurement to not reveal the group assignment.

Participant retention is promoted by the close and personal guidance by the trainers throughout the intervention period for both trial groups and the prospect of a gift card to be received at the post-intervention measurement. Reasons for non-retention is logged by the researcher.

To ensure standardized data collection and to promote data quality, the procedure for each measurement occasion is described in a standard operation plan, which is used to train the primary investigator and assistant assessors. To promote complete and replicable data sets, the data or data identifiers are recorded in a data collection form.

### Outcome measures

Primary and secondary outcome variables are measured at baseline and post-intervention and are used to determine the trial outcomes, which are the between group differences in the mean change from baseline at post-intervention in these outcome variables. Population descriptives are used to characterize both study groups at baseline. Other outcome variables relate to adverse events and exercise adherence. Each variable is evaluated in both groups, except for exercise adherence.

#### Primary outcome variable

The outcome variable to examine the primary aim is maximum gait speed. Maximum gait speed reflects propulsive capabilities and is able to discriminate between older adults with and without a fall history [12] and between older adults with and without increased fall risk [13]. Maximum gait speed is defined as the gait speed while walking at fast walking speed (“like having to catch the bus, but not running”). Maximum gait speed is assessed using the marker-based motion capture analysis that is also used to obtain foot and ankle biomechanics, which is elaborated hereafter.

#### Secondary outcome variables

##### Foot muscle’s morphology

Ultrasound is used to assess the morphology of intrinsic and extrinsic foot flexor muscles, reflecting their capacity, and was previously used to understand foot function in younger populations [37–39]. Ultrasonography is found to be a valid instrument to measure muscle size of lower extremity muscles in older adults [40].

The ultrasound scans for the assessment of foot muscle morphology are performed by the primary investigator who has extensive experience in scanning these tissues in older adults. In previous research, the reliability and measurement error of these measurements were found to be adequate to detect group mean hypertrophy in older adults as a response to training [41].

A reliable ultrasound protocol [41], modified from Crofts et al. [42] is used to measure the thickness and cross-sectional area of foot muscles using a portable ultrasound device with a 4–12 MHz linear array transducer (Philips Ultrasound, Lumify). The thickness is assessed for abductor hallucis (AbH), flexor digitorum brevis (FDB), quadratus plantae (QP), flexor hallucis brevis (FHB), abductor digiti minimi (AbDM), tibialis anterior (TA), peroneus longus together with the peroneus brevis (PER), and flexor hallucis longus (FHL). In addition, the cross-sectional area is assessed for ABH and FDB. The protocol [41] prescribes the participant’s pose and the scanning procedure. Three cine-loops are made for each muscle with repositioning of the transducer, followed by a single segmentation per scan.

ImageJ software (National Institute for Health, United States) is used for the offline segmentation of the scans. To measure the thickness and cross-sectional area of the muscle, a best quality still image is selected from the cine-loop. The thickness of a tissue is represented by the perpendicular distance between the epimysia. The cross-sectional area includes all muscle tissue of the muscle of interest that is visible on the image. The mean of three trials for each measurement is taken for further analysis.

##### Foot and ankle biomechanics during gait

**Fig. 4:**
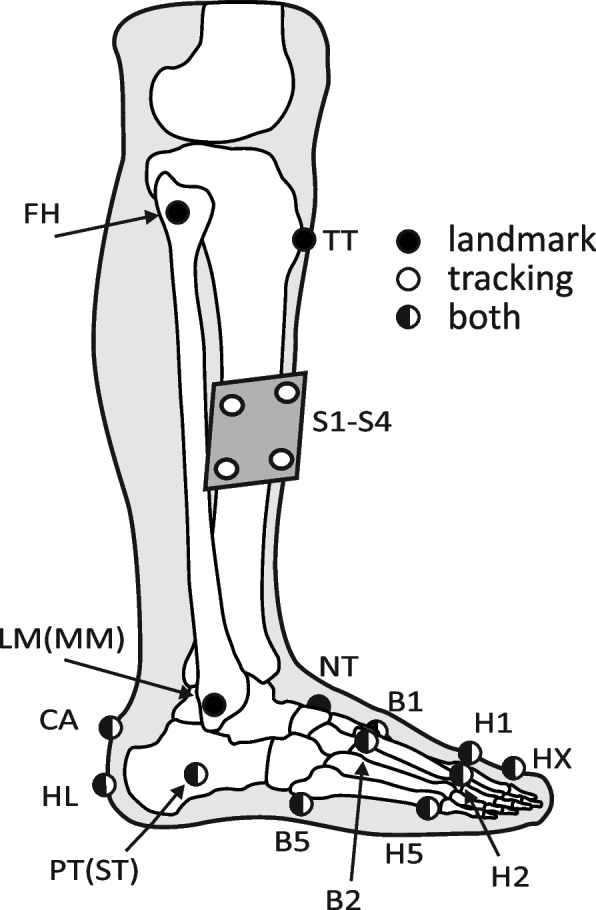
Location of the cluster markers, pointed landmarks and landmark markers. The marker configuration is a combination of that proposed by the kinematic Rizzoli foot model [43, 44] and the kinetic foot model of Bruening et al. [45]. The figure key differentiates between markers used to define the anatomical model in the static pose, markers used to track the segments’ motion in the gait trials, and markers used for both. Hidden medial markers (i.e., ST and MM) are indicated in parentheses behind their lateral counterparts. The full descriptions of the abbreviations (landmarks) are provided in Table 2. This figure is re-used from Bruening et al. [45], with permission from Elsevier

Foot and ankle kinematics and kinetics are assessed during walking at comfortable walking speed (“like walking in the park”) using a 3-dimensional (3D) marker-based motion capture system (Codamotion Ltd.; 4 CX1 units, 100 Hz) time synchronized with a recessed force plate (Advanced Mechanical Technology, Inc., OR 6–7, 1000 Hz). In accordance with the modified kinematic Rizzoli foot model [43, 44], and with the addition of landmarks from the kinetic foot model of Bruening et al. [45], 16 anatomical landmarks are identified on the lower leg and the foot, which is shown in Fig. 4 and Table 2. A four-marker pointer stick is used to locate four of these landmarks (medial malleolus, lateral malleolus, tibial tuberosity and femoral head), whereas individual markers are placed directly on the other 12 landmarks.

**Table 2:** Description of markers. These are used to define the segments of the anatomical model in the static pose and to track these segments’ motion in the gait trials

Markers / landmarks	Description
*Cluster markers*	
S1-4	Cluster on the shank with 4 markers
*Pointed landmarks*	
TT	Most anterior prominence of the tibial tuberosity
FH	Most proximal apex of the fibular head
MM	Distal apex of the medial malleolus
LM	Distal apex of the lateral malleolus
*Landmark markers*	
CA	Upper central ridge of the calcaneus posterior surface, i.e. Achilles' tendon attachment
HL	Most distal point of attachment area of the Achilles tendon on the calcaneus
ST	Most medial apex of the sustentaculum tali
PT	Lateral apex of the peroneal tubercle
NT	Most medial apex of the navicular tuberosity
B1	First metatarsal base, dorso-medial aspect of the first metatarso-cuneiform joint
H1	First metatarsal head, dorso-medial aspect of the first metatarso-phalangeal joint
HX	Most distal and dorsal point of the head of the proximal phalanx of the hallux
B2	Second metatarsal base, dorso-medial aspect of the second metatarso-cuneiform joint
H2	Second metatarsal head, dorso-medial aspect of the second metatarso-phalangeal joint
B5	Fifth metatarsal base, dorso-lateral aspect of the fifth metatarso-cuboid joint
H5	Fifth metatarsal head, dorso-lateral aspect of the fifth metatarso-phalangeal joint
*Virtual markers*	
SK_prox_	Projection of TT on the plane passing through LM, IM and FH
IM	Intermedius malleoli, midpoint between MM and LM
CA_proj_	Projection of CA target onto the ground during the static standing pose, tracked in the calcaneus reference frame
CU	Point at 2/3 of the distal distance between PT and B5 [46]
MTC	Midtarsal joint center, midpoint between CU and NV [45]
MPC	First metatarsophalangeal joint center, projection of H1 vertically ½ distance to the floor [45]
HAL_dist_	Projection of HX vertically ½ distance to the floor [45]
MET_dist_	Projection of H2 vertically ½ distance to the floor [45]
FT_dist_	Projection of H2 on the plane passing through CA, H1 and H5

The landmarks conform to the Rizzoli foot model [43, 44] unless referred to Bruening et al. [45] or Deschamps et al. [46]

After several practice trials and recording a static standing pose, used to create the anatomical model, 5 good trials are recorded using a 5-step protocol to minimize fatigue. A good trial is defined as when only the measured foot is in full contact with the force plate. Spatiotemporal parameters and joint kinematics and kinetics are obtained during the stance phase using Visual 3D software (C-Motion, Inc.). Missing marker data are handled by interpolating the data with a 3th order polynomial function. Marker data and ground reaction force data are filtered by applying a Butterworth filter (6 Hz and 20 Hz cut-off frequency, respectively). Initial contact and toe-off are determined by a vertical ground reaction force threshold of 10 N.

Segment reference frames are created for the shank and the 4 foot segments (i.e., rearfoot, midfoot, metatarsus, hallux). The shank is modelled according to the kinematic Rizzoli foot model [43], which is suitable for our kinetic purpose. The foot model is based on the kinetic foot model of Bruening et al. [45, 47], but divides the forefoot segment into a midfoot and a metatarsus segment according to the 4-segment foot model proposed by Deschamps et al. [46]. As such, our model has 4 joint centers: ankle (midpoint between medial and lateral malleoli), Chopart joint (midpoint between navicular and cuboid bone), Lisfranc joint (second metatarsal base) and first metatarsophalangeal (MTP1) joint (vertical projection first metatarsal head ½ distance to floor). The segments are modeled as cones of which the radii and masses are presented in Table 3 together with the exact segment definitions and the markers used to track the segments during the gait trials. Inertial properties are set to Visual 3D’s default values [48]. The model also contains a zero-mass dummy segment linking the calcaneus to the shank, such that it places the ankle joint center in the correct position [47]. Segment reference frames are oriented with the mediolateral axis pointing laterally to the right side of the body, the anteroposterior axis pointing forwards and the inferosuperior axis pointing upwards.

**Table 3:** The anatomical model’s segment definitions, segment properties and the markers used to track the segments’ motion

Segment	Primary axis	Extra target in primary plane	Tracking markers	Proximal radius	Distal radius	Proportion of body mass
Shank	SK_prox_ – IM [44]	LM [44]	S1-S4 [47]	0.5 (|SK_prox_ -FH|)	0.5 (|LM-MM|)	0.0465 [49]
Calcaneus	HL – MTC [45]	CA [47]	HL, PT, ST [47]	0.5 (|LM-MM|) [47]	0.5 (|LM-MM|) [47]	0.0145 [49] 0.3 [46]
Midfoot	MTC – B2 [46]	TN	B2, TN, B5	0.5 (|TN-CU|)	0.5 (|B5-B1|)	0.0145 [49] 0.3 [46]
Metatarsus	B2 – MET_dist_ [46]	H2	B1, H2, B5	0.5 (|B5-B1|)	0.5 (|H5-H1|)	0.0145 [49] 0.3 [46]
Hallux	MPC – HAL_dist_ [45]	H1 [47]	HAL_dist_, H1, HX	(|HX – HAL_dist_|) [47]	(|HX – HAL_dist_|) [47]	0.0145 [49] 0.1 [46]
Foot	CA – FT_dist_ [44]	H1 [44]	CA, H1, H5	0.5 (|PT – ST|)	0.5 (|H5 – H1|)	0.0145 [49]

The full descriptions of the abbreviations (markers/landmarks) are provided in Table 2. The shank and foot segments are modelled according to the Rizzoli foot model [44], the calcaneus and hallux segments are modelled according to Bruening et al. [45] and the midfoot and metatarsus segments are modelled according to Deschamps et al. [46]. Specifications without citations indicate that these are lacking in the literature and are sensibly determined by the authors of the current paper

Joint motions during the gait trials are obtained from the orientation of the distal segment with reference to the proximal segment, allowing 6 degrees of freedom, using the Cardan rotation order flexion/extension, abduction/adduction, and internal/ external rotation. In addition, to assess mediolongitudinal foot arch (MLA) integrity during gait, the MLA is defined as the angle between two linked line segments (i.e., CA_proj_-NT, NT-H1) projected on the sagittal plane of the foot segment [43] (see Table 3 for the foot segment definition).

Joint kinetics are calculated through inverse dynamics. The sagittal net internal ankle, Chopart, Lisfranc and MTP1 joint moment are calculated in the proximal segment’s reference frame. In addition, the power in these joints is derived as the scalar dot product of the joint moment and angular velocity. Joint kinetics are only considered once the net internal sagittal joint moments are negative (i.e., once the CoP has passed the distal end of the joint’s proximal segment in the anterior direction [10]). Kinetic variables are normalized to body weight.

The obtained stance phase kinematic and kinetic outcome variables for each trial are MLA deformation (i.e., change in MLA from initial contact to its maximum), MLA recoil (i.e., change in MLA from its maximum to toe-off), peak sagittal internal joint moment (ankle, Chopart, Lisfranc, and MTP1 joint), peak positive scalar joint power (ankle, Chopart, Lisfranc, MTP1 joint) and peak negative scalar joint power (Chopart, Lisfranc, MTP1 joint). Each outcome variable is averaged over the trials.

##### Gait speed

Preferred gait speed is assessed during the comfortable walking speed trials described above. Maximum gait speed is assessed in five additional trials in which the participants are instructed to walk as fast as possible over the walkway (“like having to catch the bus, without running”). For both conditions, gait speed is calculated as the rate of change in heel marker position along the anteroposterior axis of the lab coordinate system between two consecutive foot strikes of the ipsilateral foot. These foot strike events are identified as the frames where the sagittal velocity of the heel marker drops below 500 mm/s [50, 51].

##### Balance during gait

Balance during gait is examined through calculating the lateral margin of stability [52]. This variable quantifies stability in dynamic situations by relating the body’s center of mass (CoM) to the center of pressure (CoP) [52], while accounting for the velocity of the CoM and considering the human body as an inverted pendulum [52]. To this end, participants perform 5 additional gait trials at preferred walking speed while the body’s contour is tracked using 8 video camera’s (Qualisys AB, Miqus video, 50 Hz) time synchronized with the collection of the ground reaction force (Advanced Mechanical Technology, Inc., OR6-7, 1000 Hz). The remainder of the procedure is as described before. The video recordings are post-processed using Theia software (Theia Markerless, Inc., Theia3D) to result in the position of the CoM. The extrapolated center of mass, a quantity needed to obtain the margin of stability, is calculated for each time instance as the position of the CoM, plus its velocity times a factor equal to $\sqrt{^{l}\!\left/ \!_{g}\right.}$, where $l$ is the maximum height of the CoM and $g$ is the gravitational acceleration. The margin of stability is defined as the minimum lateral distance between the extrapolated center of mass and the mean CoP position during single leg stance [53]. Contralateral foot-off and foot strike are defined as when the sagittal velocity of the model’s distal end of the toes’ segment exceeds 500 mm/s and the heel marker drops below 500 mm/s, respectively [50, 51]. The margin of stability is averaged over the trials.

##### Step length

The step length is derived from the gait analysis used to assess balance during gait. Step length is defined as the distance between the contralateral heel landmark position at foot strike and the following ipsilateral heel landmark position at the force plate hit along the lab’s anteroposterior axis.

##### Isometric toe flexor strength

To assess toe flexor strength, the participant is asked to stand on both feet, hip width apart, with one foot on the pressure plate (Materialise NV). The participant is then verbally encouraged to push down as hard as possible for the duration of approximately 3 s with either the hallux or the lesser toes, while the entire foot remains on the floor [54, 55]. Movement of the other toes is allowed. The upper body is kept in an upright position and the knees near maximally extended, which is visually inspected by the assessor. Both test conditions are practiced once and completed three times in alternated fashion with a rest period of 30 s between the trials. The automated zone divisions are manually corrected afterwards (Materialise NV, Footscan v9). The peak force under each of both plantar regions is normalized to body weight and averaged over 3 trials.

##### Self-reported mobility limitations

As a proxy of fall risk [17, 56], the participant is asked a single question about whether or not experiencing difficulties with mobility, gait or balance in daily life.

##### Physical activity engagement

The participant is asked to weekly report in the diary (see below) the time spent in physical activities in bouts of at least 10 min duration that is experienced by the participant at least as moderate intense (≥ 5 on a 10-point scale of how hard one feel he or she is exercising) [57].

##### Fall incidents during intervention period

The participant is asked to report any fall incidents that occurs during the intervention period in their diary (see below). A fall is defined according to the Prevention of Falls Network Europe, as ‘an unexpected event in which the subject comes to rest on the ground, floor, or lower level’ [58]. In case of a fall, the participant is inquired about the circumstances (i.e., what, how and when) and the consequences (e.g., injuries) of the fall incident. The number of falls is also documented.

##### Fear of falling

Fear of falling is assessed by the Fall Efficacy Scale-International (FES-I) [59] and is found to be associated with gait modifications [60]. The FES-I is a 16-item questionnaire in which an individual grades his concern about falling during various activities on a 4-point scale, resulting in a total score ranging from 16 to 64. A higher score indicates a greater concern. The Dutch translation of FES-I that is used in this study has acceptable reliability and validity [61].

##### Physical functioning

Performance on gait speed, balance and lower extremity strength is assessed using the Short Physical Performance Battery (SPPB) [62]. The SPPB is a widely used test in the older population and the outcome is predictive of a variety of health outcomes. More specifically, SPBB score was shown to be associated with falling [63]. The SPPB consists of 5 short tests: 3 10-s balance test (i.e., double leg stance, semi-tandem stance, tandem stance), 4-m walk test and a timed chair-stand-test. The test results in a score, ranging from 0 to 12. A higher score indicates better performance.

#### Population descriptives

##### Demographics

The demographics age, gender and living situation (i.e., dependent/independent, living together/alone) are reported.

##### Body length and weight

Body length and weight are assessed using a digital stadiometer (Dong Sahn Jenix co., DS-103).

##### Mobility related conditions

The participant is asked about the use of walking aids, uncorrected visual impairments, uncorrected hearing loss, musculoskeletal and neurological conditions, use of medicines, presence of dizziness, and number of falls over the past 12 months. Additionally, the dorsal flexion passive range of motion at the hallux metatarsophalangeal joint is assessed and hallux valgus is graded using the Manchester scale [64–66]. Protective sensibility of the plantar side of the foot is evaluated according to the Dutch guideline for the diabetic foot (2017) with a 10-g Semmes–Weinstein monofilament.

##### Cognitive functioning

Cognitive functioning is assessed by the Montreal Cognitive Assessment (MoCA), addressing 8 domains of cognitive functioning [67]. It exhibits good reliability and validity [67] and it was shown to be superior compared to the Mini-Mental State Examination in distinguishing among a group of individuals with mild cognitive impairment showing less ceiling effects [68]. The maximum score is 30, indicating maximum cognitive functioning.

##### Health related quality of life

The 36 item Short Form Health Survey (SF-36) is used to assess health related quality of life [69]. The survey addresses 8 domains, among which physical functioning and mental health, both being related to balance and gait. The item scores are transformed such that a higher score indicates better health. Total scores and the scores on the physical functioning domain and mental health domain are expressed as a score out of 100. The Dutch translation of the SF-36 used in this study proved to be a reliable and valid instrument for the general population [70].

##### Hand grip strength

Hand grip strength is measured using a hydraulic hand grip dynamometer (Baseline, 12–0241 LiTE). Hand grip dynamometry, reliable for measuring grip strength in older adults [71], is proposed a fundamental element of physical examination of older adults [72]. The participant, seated and having the dominant arm rested on a table with the elbow in 90 degrees of flexion, is encouraged to exert maximal grip strength. After a practice trial, the maximum force of one trial is recorded.

##### Physical activity behavior

A physical activity monitor (ActivPAL, PAL Technologies Ltd.) [73, 74] is used to record physical activity 24 h a day for a maximum of 7 days between the home visit and the baseline laboratory session. This wearable is used to obtain average daily time spent sedentary, standing and stepping (i.e., cycling and walking). In addition, the average daily stepping time with cadence ≥ 75 steps per minute in bouts of at least 10 min is obtained [46].

##### Characteristics of the functional exercise program

The setting (e.g., physiotherapy practice, senior gym class) and the weekly frequency and duration of the functional exercise program in which the participant is involved is documented. Also weekly, it is verified whether the participant is still involved in the functional exercise program.

#### Other outcome variables

##### Movement related discomfort

The participants in the PIFM strengthening training group weekly report movement related discomfort experienced during the training in their diary. Both the PIFM strengthening training group and the control group weekly report movement related discomfort experienced throughout the week (outside the training) in their diary enabling the comparison between the groups for the occurrence of adverse events.

##### Exercise adherence

The participant notes the completion of each unsupervised training session in the diary. The attendance to the supervised session is registered by the trainer. Overall adherence to the training is expressed in the number of completed sessions as a percentage of the total number of prescribed training sessions.

### Statistical analysis

All statistical analyses are completed using SPSS 28.0 (IBM) software. Baseline and post-intervention data, as well as changes from baseline, are summarized per group by descriptive statistics (i.e., means and standard deviations for continuous variables and absolute and relative frequencies for categorical variables). In addition, the between group differences in mean change from baseline are presented with its 95% confidence interval.

Missing data are explored in terms of numbers and characteristics. If the proportion of missing data is below 5% or when the missing data occurs completely at random, complete case analysis is performed. If these criteria are not met, multiple imputation is used to handle the missing data [75].

The primary analyses test the hypotheses that the mean change from baseline in the primary outcome variable (maximum gait speed) and secondary outcome variables is superior for the intervention group compared to the control group. To this end, the data for each participant that completed the post-intervention measurements are included as randomized to perform an intention-to-treat analysis. An additional per-protocol analysis is conducted, including only the participants from the PIFM strengthening training group who completed ≥ 75% of the prescribed session. Analyses of covariance (ANCOVA) are performed with the baseline value of the outcome variable included as covariate [76, 77], after having checked the assumptions (i.e., no outliers, normal distribution of dependent variable, independency covariate and treatment effect, linear relationship between covariate and dependent variable, homogeneity of regression slopes, homogeneity of variance of dependent variable). Additionally, in separate linear regression analyses, the potential modification of the intervention effect is explored for the potential modifiers ‘change from baseline in physical activity’, ‘change from baseline in muscles’ morphology’ and ‘change from baseline in isometric toe flexor strength’. α = 0.05 (one-tailed) is applied to draw conclusions on the statistical analyses.

Variables designated as ‘other outcome variables’ are analyzed using a descriptive approach.

## Discussion

The proposed protocol presents the rational and methodology for an RCT to investigate the effect of a PIFM strengthening training on mobility related outcome variables in older adults who are involved in a group-based functional exercise program. This responds to the identified need for a high-quality study on the effect of such a training on outcome variables that are meaningful to this target population in the sense of decreasing fall risk [18].

We have strived for high-quality methodology by randomization and concealed allocation, blinding of assessors, collecting confounders and maximizing adherence and retention. Although we make substantial effort to blind the assessors, it is not inconceivable that the group allocation is revealed by either the participant at the post-intervention measurement or the trainer, who is in close contact with the primary investigator. The guess of the allocation after the post-intervention measurement will show whether assessor blinding was accomplished successfully.

Another internal validity limitation relates to the contrast between the trial arms. Ideally, any effect may be ascribed to the PIFM strengthening training. However, because it is impossible to blind participants in an exercise program, related bias may occur [78]. In addition, participating in the PIFM strengthening training may promote physical activity, among which attendance to the functional exercise group. We attempted to minimize this bias by introducing the diary for the control group, containing similar questions related to mobility and physical activity. Nevertheless, we included physical activity as a secondary outcome variable. This enables us to evaluate the confounding effect of change from baseline in physical activity on change from baseline in other outcome variables.

The design of the PIFM strengthening training took into account factors to optimize the intervention effect. Some of these factors correspond well to clinical practice whereas others do less. The progressive nature agrees with the principle of overload, which is common practice in physical therapy and is recommended for functional training to prevent falling [6]. In addition, the training is delivered and supervised by a 4th year physiotherapy student who is able to properly provide instructions and coach the participant to adhere to the training, similar to the practice of fall preventive exercise programs. However, the intensity of the training, targeting primarily the feet, 5 times a week for 20 min, is not common practice. This may compromise generalizability to existing fall preventive exercise programs, which usually involve a weekly 1-h session in which all muscle groups are addressed. Nevertheless, knowing that a 12-week intensive PIFM strengthening training can improve mobility may advocate the integration of such exercises in fall preventive and ongoing exercise programs.

Some other factors also endorse the external validity. Regarding the population, we decided to recruit the participants in the target setting, which is in fall preventive or functional exercise programs. Additionally, we adopted broad eligibility criteria, which further supports the generalizability of the findings to the target population. Regarding the intervention, the training consists of both resistance and functional exercises, which seems to be most effective to prevent falling in older adults [7].

The results of this RCT give guidance relating PIFM strengthening to existing fall preventive exercise programs. To this end, all training materials (i.e., written instructions, training guide and the instructional videos) will be made available once the intended peer-reviewed article has been published open access.

## Supplementary Information

Additional file 1. SPIRIT checklist.

Additional file 2. CONSORT 2017 Checklist for Nonpharmacologic Treatments.

Additional file 3. Training guide.

Additional file 4. Trainer’s guide.

Additional file 5. Diary (control).

## Data Availability

The datasets generated and/or analysed during the current study are not publicly available but are available from the corresponding author on reasonable request.

## Abbreviations

MTP1: Hallux’ metatarsal phalangeal joint
PIFM: Plantar intrinsic foot muscle
RCT: Randomized controlled trial
STIFF: STrengthening the Intrinsic Foot Flexor muscles
