# Supplementary material for: Effects of a 12-week intrinsic foot muscle strengthening training (STIFF) on gait in older adults: a parallel randomized controlled trial protocol
Source: BMC Sports Sci Med Rehabil. 2024 Jul 20;16:158. doi: 10.1186/s13102-024-00944-z (PMC11542310; doi:10.1186/s13102-024-00944-z)
Supplement: Supplementary file 2 — Additional file 2. CONSORT 2017 Checklist for Nonpharmacologic Treatments. [file 13102_2024_944_MOESM2_ESM.doc]

2017 CONSORT Checklist of Information to Include When Reporting Randomized Trials Assessing Nonpharmacologic Treatment (1)

| Section/Topic | Item No | Checklist item | Reported on page No |
| --- | --- | --- | --- |
| Title and abstract | | | |
|  | 1a | Identification as a randomised trial in the title | 1 |
|  | 1b | Structured summary of trial design, methods, results, and conclusions  Refer to CONSORT extension for abstracts for NPT trials | 1 |
| Introduction | | | |
| Background and objectives | 2a | Scientific background and explanation of rationale | 3-4 |
|  | 2b | Specific objectives or hypotheses | 4 |
| Methods | | | |
| Trial design | 3a | Description of trial design (such as parallel, factorial) including allocation ratio  When applicable, how care providers were allocated to each trial group | 4 |
|  | 3b | Important changes to methods after trial commencement (such as eligibility criteria), with reasons | n/a |
| Participants | 4a | Eligibility criteria for participants  When applicable, eligibility criteria for centers and for care providers | 6 |
|  | 4b | Settings and locations where the data were collected | 4-5 |
| Interventions | 5 | The interventions for each group with sufficient details to allow replication, including how and when they were actually administered  Precise details of both the experimental treatment and comparator | 19-21 |
|  | 5a | Description of the different components of the interventions and, when applicable, description of the procedure for tailoring the interventions to individual participants. | 20 |
|  | 5b | Details of whether and how the interventions were standardized. | 19-20 |
|  | 5c | Details of whether and how adherence of care providers to the protocol was assessed or enhanced | 19-20 |
|  | 5d | Details of whether and how adherence of participants to interventions was assessed or enhanced | 20 |
| Outcomes | 6a | Completely defined pre-specified primary and secondary outcome measures, including how and when they were assessed | 8-19 |
|  | 6b | Any changes to trial outcomes after the trial commenced, with reasons | n/a |
| Sample size | 7a | How sample size was determined  When applicable, details of whether and how the clustering by care providers or centers was addressed | 6-7 |
|  | 7b | When applicable, explanation of any interim analyses and stopping guidelines | n/a |
| Randomisation: |  |  |  |
| Sequence generation | 8a | Method used to generate the random allocation sequence | 7 |
|  | 8b | Type of randomisation; details of any restriction (such as blocking and block size) | 7 |
| Allocation concealment mechanism | 9 | Mechanism used to implement the random allocation sequence (such as sequentially numbered containers), describing any steps taken to conceal the sequence until interventions were assigned | 7 |
| Implementation | 10 | Who generated the random allocation sequence, who enrolled participants, and who assigned participants to interventions | 7 |
| Blinding | 11a | If done, who was blinded after assignment to interventions (e.g., participants, care providers, those administering co-interventions, those assessing outcomes) and how | 7 |
|  | 11b | If relevant, description of the similarity of interventions | n/a |
|  | 11c | If blinding was not possible, description of any attempts to limit bias | 7,8 |
| Statistical methods | 12a | Statistical methods used to compare groups for primary and secondary outcomes  When applicable, details of whether and how the clustering by care providers or centers was addressed | 21-22 |
|  | 12b | Methods for additional analyses, such as subgroup analyses and adjusted analyses | 21 |
| Results | | | |
| Participant flow (a diagram is strongly recommended) | 13a | For each group, the numbers of participants who were randomly assigned, received intended treatment, and were analysed for the primary outcome  The number of care providers or centers performing the intervention in each group and the number of patients treated by each care provider or in each center | n/a |
|  | 13b | For each group, losses and exclusions after randomisation, together with reasons | n/a |
|  | 13c | For each group, the delay between randomization and the initiation of the intervention | n/a |
|  | New | Details of the experimental treatment and comparator as they were implemented | n/a |
| Recruitment | 14a | Dates defining the periods of recruitment and follow-up | n/a |
|  | 14b | Why the trial ended or was stopped | n/a |
| Baseline data | 15 | A table showing baseline demographic and clinical characteristics for each group  When applicable, a description of care providers (case volume, qualification, expertise, etc.) and centers (volume) in each group | n/a |
| Numbers analysed | 16 | For each group, number of participants (denominator) included in each analysis and whether the analysis was by original assigned groups | n/a |
| Outcomes and estimation | 17a | For each primary and secondary outcome, results for each group, and the estimated effect size and its precision (such as 95% confidence interval) | n/a |
|  | 17b | For binary outcomes, presentation of both absolute and relative effect sizes is recommended | n/a |
| Ancillary analyses | 18 | Results of any other analyses performed, including subgroup analyses and adjusted analyses, distinguishing pre-specified from exploratory | n/a |
| Harms | 19 | All important harms or unintended effects in each group (for specific guidance see CONSORT for harms) | n/a |
| Discussion | | | |
| Limitations | 20 | Trial limitations, addressing sources of potential bias, imprecision, and, if relevant, multiplicity of analyses  In addition, take into account the choice of the comparator, lack of or partial blinding, and unequal expertise of care providers or centers in each group | 22-23 |
| Generalisability | 21 | Generalizability (external validity) of the trial findings according to the intervention, comparators, patients, and care providers and centers involved in the trial | 22-23 |
| Interpretation | 22 | Interpretation consistent with results, balancing benefits and harms, and considering other relevant evidence | n/a |
| Other information | | |  |
| Registration | 23 | Registration number and name of trial registry | 5 |
| Protocol | 24 | Where the full trial protocol can be accessed, if available | n/a |
| Funding | 25 | Sources of funding and other support (such as supply of drugs), role of funders | 24 |

1. Barbour V, Bhui K, Chescheir N, Clavien PA, Diener MK, Glasziou P, et al. CONSORT Statement for randomized Trials of nonpharmacologic treatments: A 2017 update and a CONSORT extension for nonpharmacologic Trial Abstracts. Ann Intern Med. 2017; doi:10.7326/M17-0046.
