# Supplementary material for: Effects of a 12-week intrinsic foot muscle strengthening training (STIFF) on gait in older adults: a parallel randomized controlled trial protocol
Source: BMC Sports Sci Med Rehabil. 2024 Jul 20;16:158. doi: 10.1186/s13102-024-00944-z (PMC11542310; doi:10.1186/s13102-024-00944-z)
Supplement: Supplementary file 3 — Additional file 3. Training guide. [file 13102_2024_944_MOESM3_ESM.pdf]

ID:

STIFF TRAINING GUIDE

For the study into foot muscles and the ability to move in older adults

Fontys Allied Health Professions
in collaboration with
SGE

Fontys

STIFF

SGE

Your participation in the study

You are participating in the study on the effect of foot training on balance and walking. For this purpose, you have been assigned to the group that will undergo the **foot training**. You can read more about this on the following pages.

It is of utmost importance for the study that the researcher, Lydia Willemse, does not know that you are involved in the foot training. Therefore, please try to keep this unrevealed when you see her at the last measurement.

We consider it very important that you report any emerging symptoms or discomfort during or outside the training to the trainer. If necessary, we will examine together whether and how you can continue the training.

! **Always report discomfort during or after training to the trainer and note this in the diary**

The timeline of the study

**MEASUREMENT 1A: HOME**		Date measurement 1A:
**MEASUREMENT 1B: FONTYS**		Date measurement 1B:
4 x self training<br>1 x with trainer	**WEEK 1**	Date with trainer:<br>Location:
4 x self training<br>1 x with trainer	**WEEK 2**	Date with trainer:<br>Location:
4 x self training<br>1 x with trainer	**WEEK 3**	Date with trainer:<br>Location:
4 x self training<br>1 x with trainer	**WEEK 4**	Date with trainer:<br>Location:
4 x self training<br>1 x with trainer	**WEEK 5**	Date with trainer:<br>Location:
4 x self training<br>1 x with trainer	**WEEK 6**	Date with trainer:<br>Location:
4 x self training<br>1 x with trainer	**WEEK 7**	Date with trainer:<br>Location:
4 x self training<br>1 x with trainer	**WEEK 8**	Date with trainer:<br>Location:
4 x self training<br>1 x with trainer	**WEEK 9**	Date with trainer:<br>Location:
4 x self training<br>1 x with trainer	**WEEK 10**	Date with trainer:<br>Location:
4 x self training<br>1 x with trainer	**WEEK 11**	Date with trainer:<br>Location:
4 x self training<br>1 x with trainer	**WEEK 12**	Date with trainer:<br>Location:
**MEASUREMENT 2: FONTYS**		

Location Fontys:
Rachelsmolen 1
Building R12
5612 MA Eindhoven

The foot training

The foot training consists of a **12-week** program. Each week you will train 4 x 20-30 minutes at home without a trainer (it may take a little more time in the beginning) and 1 x 30 minutes with a trainer.

For the **training with the trainer**, you can choose whether you want to receive the trainer at home or you visit us at Fontys Allied Health Professions where you can train with other participants. The training will be given by a 4th year physical therapy student. On the previous page you will find the contact information. The trainer will discuss with you when and where the training will take place.

You perform the **training without the trainer** by yourself using this training book and the instructional videos. Below the exercise instructions, you will find your additional instructions for performing the exercises. Here it says in which position you perform the exercise and with how many repetitions or how many seconds you should perform the exercise. When this changes, the trainer will indicate this on that page. You should always follow the bottom instructions. Your goal, unless otherwise agreed, is to do all the exercises. This takes about 20-30 minutes each session.

The image of each exercise is accompanied by a QR code that you can scan with your smartphone or tablet. This will lead you to an instructional video for each exercise. If you do not have a smartphone or tablet, that is not a problem. The trainer will discuss with you how to still access the videos. The QR code on the right will lead you to the introductory video.

When performing standing exercises, for safety, always have something nearby that you can hold onto, such as the back of a sturdy chair or the kitchen counter.

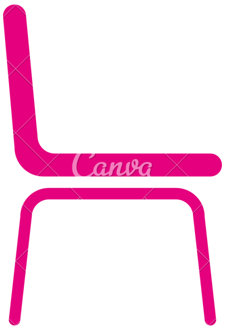

The complete foot training

Want to do all the exercises in one run using the instructional videos? Then, scan this QR code:

Training log and diary

For the study, it is important that we know how you experience the exercises. We therefore ask you to indicate on the right-hand page of the exercise book how feasible you perceived the execution of the exercise. Fill in this page each time you have performed an exercise. You do this for your left foot and right foot seperately by placing a circle around the smiley that best fits your experience.

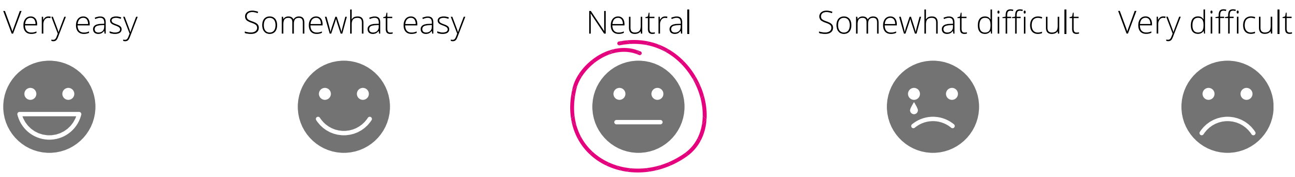

Only circle the smiley if you performed the exercise or if you attempted to perform the exercise.

At the back of the training book you will find the diary containing some more general questions. We also ask that you fill these out after each training session.

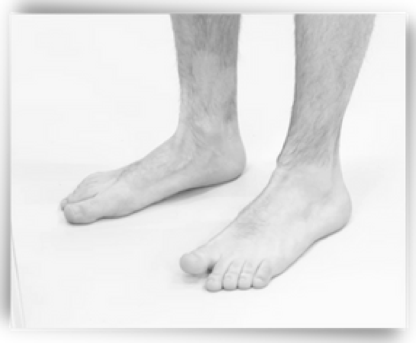

Exercise 1: Big toe and lesser toes extension

Keep the ball of the foot on the ground

Instruction:

- Place your feet flat on the floor at hip width;
- Lift the big toe of the left foot off the floor while the lesser toes remain on the floor;
- Hold this for **5 seconds**;
- Now lift the little toes up while keeping your big toe on the ground;
- Hold this for 5 seconds;
- After you have done all the repetitions, repeat everything with your other foot.

Position	Side	Repetitions	Rest	Repetitions	Moment of adjustment (week, training#)
Seated	L / R	10 x 5sec			

Exercise 1: Big toe and lesser toes extension

- Very easy
- Somewhat easy
- Neutral
- Somewhat difficult
- Very difficult

Week	Training	Feasibility LEFT	Feasibility RIGHT
1	1		
	2		
	3		
	4		
	5		
2	1		
	2		
	3		
	4		
	5		
3	1		
	2		
	3		
	4		
	5		
4	1		
	2		
	3		
	4		
	5		
5	1		
	2		
	3		
	4		
	5		
6	1		
	2		
	3		
	4		
	5		

Very easy
Somewhat easy
Neutral
Somewhat difficult
Very difficult

Exercise 1: Big toe and lesser toes extension

Week	Training	Feasibility LEFT	Feasibility RIGHT
7	1		
	2		
	3		
	4		
	5		
8	1		
	2		
	3		
	4		
	5		
9	1		
	2		
	3		
	4		
	5		
10	1		
	2		
	3		
	4		
	5		
11	1		
	2		
	3		
	4		
	5		
12	1		
	2		
	3		
	4		
	5		

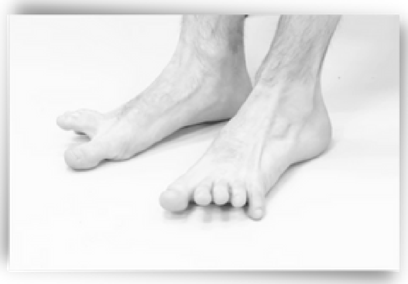

Exercise 2: Toe spread and squeeze

Instruction:

- Sit on a chair;
- Place your feet flat on the floor at hip width;
- Lift the toes of the left foot off the floor and spread the toes as far apart as possible;
- Hold this for **5 seconds**;
- Now squeeze the toes of the same foot together;
- Hold this for **5 seconds**;
- After you have done all the repetitions, repeat everything with your right foot.

You may train both feet at the same time

Position	Side	Repetitions	Rest	Repetitions	Moment of adjustment (week, training#)
Seated	L / R	10 x 5sec			

Exercise 2: Toe spread and squeeze

- Very easy
- Somewhat easy
- Neutral
- Somewhat difficult
- Very difficult

Week	Training	Feasibility LEFT	Feasibility RIGHT
1	1		
	2		
	3		
	4		
	5		
2	1		
	2		
	3		
	4		
	5		
3	1		
	2		
	3		
	4		
	5		
4	1		
	2		
	3		
	4		
	5		
5	1		
	2		
	3		
	4		
	5		
6	1		
	2		
	3		
	4		
	5		

Exercise 2: Toe spread and squeeze

- Very easy
- Somewhat easy
- Neutral
- Somewhat difficult
- Very difficult

Week	Training	Feasibility LEFT	Feasibility RIGHT
7	1		
	2		
	3		
	4		
	5		
8	1		
	2		
	3		
	4		
	5		
9	1		
	2		
	3		
	4		
	5		
10	1		
	2		
	3		
	4		
	5		
11	1		
	2		
	3		
	4		
	5		
12	1		
	2		
	3		
	4		
	5		

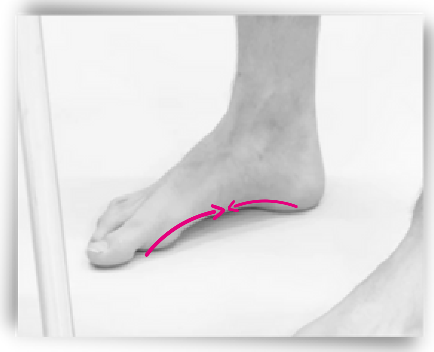

Exercise 3: Short foot exercise

Instruction:

- Check the table below to see if you perform this exercise standing or sitting;
- Place your feet flat on the floor at hip width;
- Pull the ball of the foot toward the heel, without curling the toes;
- While doing this, keep the ball of the foot and the heel on the floor;
- Hold for **3 seconds**
- After you have done all the repetitions, repeat everything with your other foot

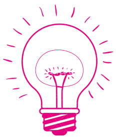

This is a tough exercise, but keep trying.

The movement is only small.

Position	Side	Repetitions	Rest	Repetitions	Moment of adjustment (week, training#)
Seated	L / R	10 x 3sec			

Exercise 3: Short foot exercise

- Very easy
- Somewhat easy
- Neutral
- Somewhat difficult
- Very difficult

Week	Training	Feasibility LEFT	Feasibility RIGHT
1	1		
	2		
	3		
	4		
	5		
2	1		
	2		
	3		
	4		
	5		
3	1		
	2		
	3		
	4		
	5		
4	1		
	2		
	3		
	4		
	5		
5	1		
	2		
	3		
	4		
	5		
6	1		
	2		
	3		
	4		
	5		

Exercise 3: Short foot exercise

Very easy
Somewhat easy
Neutral
Somewhat difficult
Very difficult

Week	Training	Feasibility LEFT	Feasibility RIGHT
7	1		
	2		
	3		
	4		
	5		
8	1		
	2		
	3		
	4		
	5		
9	1		
	2		
	3		
	4		
	5		
10	1		
	2		
	3		
	4		
	5		
11	1		
	2		
	3		
	4		
	5		
12	1		
	2		
	3		
	4		
	5		

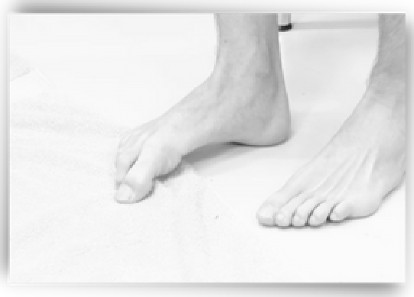

Exercise 4: Towel curl

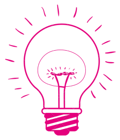

This is a tough exercise, but keep trying.

Equipment:

- Towel

Instruction:

- Sit on a chair;
- Place the towel in front of you on the floor;
- Place your feet flat on the floor at hip width;
- With the toes of one foot, curl up the towel a little bit and hold for **2 seconds**;
- Release the towel to grab the towel a little further;
- After you have done all the repetitions, repeat everything with your other foot

Position	Side	Repetitions	Rest	Repetitions	Moment of adjustment (week, training#)
Seated	L / R	5 x 2 sec			

Exercise 4: Towel curl

- Very easy
- Somewhat easy
- Neutral
- Somewhat difficult
- Very difficult

Week	Training	Feasibility LEFT	Feasibility RIGHT
1	1		
	2		
	3		
	4		
	5		
2	1		
	2		
	3		
	4		
	5		
3	1		
	2		
	3		
	4		
	5		
4	1		
	2		
	3		
	4		
	5		
5	1		
	2		
	3		
	4		
	5		
6	1		
	2		
	3		
	4		
	5		

Exercise 4: Towel curl

- Very easy
- Somewhat easy
- Neutral
- Somewhat difficult
- Very difficult

Week	Training	Feasibility LEFT	Feasibility RIGHT
7	1		
	2		
	3		
	4		
	5		
8	1		
	2		
	3		
	4		
	5		
9	1		
	2		
	3		
	4		
	5		
10	1		
	2		
	3		
	4		
	5		
11	1		
	2		
	3		
	4		
	5		
12	1		
	2		
	3		
	4		
	5		

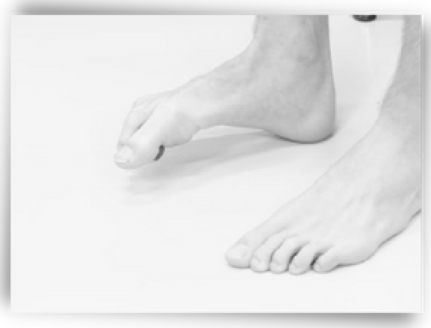

Exercise 5: Marble pick up

This is a tough exercise, but keep trying.

Equipment:

- Marble or else a cap, eraser or similar object.

Instruction:

- Sit on a chair;
- Put your feet flat on the floor at hip width;
- Place the object in front of you on the floor;
- Grasp the object with your toes and hold for **2 seconds**;
- Release the object again;
- After you have done all the repetitions, repeat everything with your other foot.

Position	Side	Repetitions	Rest	Repetitions	Moment of adjustment (week, training#)
Seated	L / R	5 x 2 sec			

Exercise 5: Marble pick up

- Very easy
- Somewhat easy
- Neutral
- Somewhat difficult
- Very difficult

Week	Training	Feasibility LEFT	Feasibility RIGHT
1	1		
	2		
	3		
	4		
	5		
2	1		
	2		
	3		
	4		
	5		
3	1		
	2		
	3		
	4		
	5		
4	1		
	2		
	3		
	4		
	5		
5	1		
	2		
	3		
	4		
	5		
6	1		
	2		
	3		
	4		
	5		

Exercise 5: Marble pick up

- Very easy
- Somewhat easy
- Neutral
- Somewhat difficult
- Very difficult

Week	Training	Feasibility LEFT	Feasibility RIGHT
7	1		
	2		
	3		
	4		
	5		
8	1		
	2		
	3		
	4		
	5		
9	1		
	2		
	3		
	4		
	5		
10	1		
	2		
	3		
	4		
	5		
11	1		
	2		
	3		
	4		
	5		
12	1		
	2		
	3		
	4		
	5		

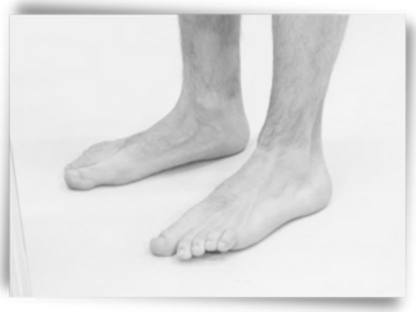

Exercise 6: Big toe and lesser toes press

Instruction:

- Stand on two legs;
- Place your feet flat on the floor at hip width;
- Push the big toe against the floor as firm as possible without curling the toes;
- Hold this for **5 seconds**;
- Now push the smaller toes into the ground as firm as possible;
- Hold this for **5 seconds**;
- Do this for the number of repetitions stated below.

When you start cramping, stop for a moment.

Stretching the toes may help reduce cramping

Position	Side	Repetitions	Rest	Repetitions	Moment of adjustment (week, training#)
Seated	L / R	10 x 5 sec			

Exercise 6: Big toe and lesser toes press

- Very easy
- Somewhat easy
- Neutral
- Somewhat difficult
- Very difficult

Week	Training	Feasibility LEFT	Feasibility RIGHT
1	1		
	2		
	3		
	4		
	5		
2	1		
	2		
	3		
	4		
	5		
3	1		
	2		
	3		
	4		
	5		
4	1		
	2		
	3		
	4		
	5		
5	1		
	2		
	3		
	4		
	5		
6	1		
	2		
	3		
	4		
	5		

Very easy
Somewhat easy
Neutral
Somewhat difficult
Very difficult

Exercise 6: Big toe and lesser toes press

Week	Training	Feasibility LEFT	Feasibility RIGHT
7	1		
	2		
	3		
	4		
	5		
8	1		
	2		
	3		
	4		
	5		
9	1		
	2		
	3		
	4		
	5		
10	1		
	2		
	3		
	4		
	5		
11	1		
	2		
	3		
	4		
	5		
12	1		
	2		
	3		
	4		
	5		

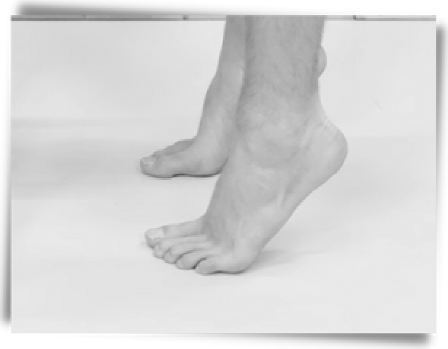

Exercise 7: Heel raise

Instruction:

- Check the table below to see in which position you perform this exercise;
- Place your feet flat on the floor at hip width;
- Then lift your heel as high as you can while keeping your toes on the ground;
- Hold for **2 seconds**;
- Now slowly lower your heel(s) until the foot (feet) are flat on the floor again;
- Did you perform this exercise on one leg? Then repeat everything with your other foot on the floor.

Like grabbing something off a high shelf

You may perform this exercise anywhere and anytime

Position	Side	Repetitions	Rest	Repetitions	Moment of adjustment (week, training#)
Seated	L / R	10 x 2 sec			

Exercise 7: Heel raise

- Very easy
- Somewhat easy
- Neutral
- Somewhat difficult
- Very difficult

Week	Training	Feasibility LEFT	Feasibility RIGHT
1	1		
	2		
	3		
	4		
	5		
2	1		
	2		
	3		
	4		
	5		
3	1		
	2		
	3		
	4		
	5		
4	1		
	2		
	3		
	4		
	5		
5	1		
	2		
	3		
	4		
	5		
6	1		
	2		
	3		
	4		
	5		

Exercise 7: Heel raise

- Very easy
- Somewhat easy
- Neutral
- Somewhat difficult
- Very difficult

Week	Training	Feasibility LEFT	Feasibility RIGHT
7	1		
	2		
	3		
	4		
	5		
8	1		
	2		
	3		
	4		
	5		
9	1		
	2		
	3		
	4		
	5		
10	1		
	2		
	3		
	4		
	5		
11	1		
	2		
	3		
	4		
	5		
12	1		
	2		
	3		
	4		
	5		

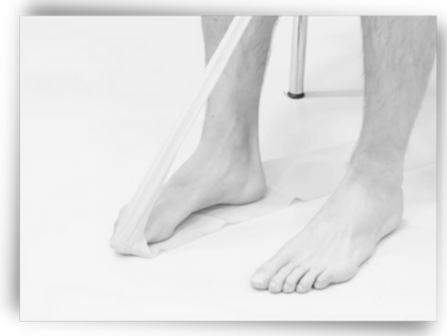

Exercise 8: big toe flexion against resistance

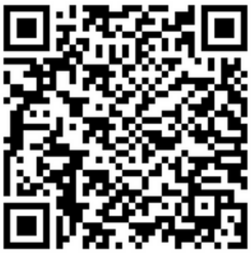

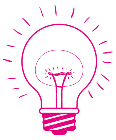

The more tension on the band, the harder the exercise

Keep the ball of your foot on the floor

Equipment:

- Resistance band

Instruction:

- Sit on a chair and place your feet flat on the floor at hip width;
- Place the resistance band under your foot with one end under the heel and the other around the big toe
- Stand up with the band in your hand to create tension;
- Push the big toe into the band;
- Hold this for **5 seconds**;
- After you have done all the repetitions, repeat everything with your other foot.

Position	Side	Repetitions	Rest	Repetitions	Moment of adjustment (week, training#)
Seated	L / R	10 x 5 sec			

Exercise 8: big toe flexion against resistance

- Very easy
- Somewhat easy
- Neutral
- Somewhat difficult
- Very difficult

Week	Training	Feasibility LEFT	Feasibility RIGHT
1	1		
	2		
	3		
	4		
	5		
2	1		
	2		
	3		
	4		
	5		
3	1		
	2		
	3		
	4		
	5		
4	1		
	2		
	3		
	4		
	5		
5	1		
	2		
	3		
	4		
	5		
6	1		
	2		
	3		
	4		
	5		

Exercise 8: big toe flexion against resistance

- Very easy
- Somewhat easy
- Neutral
- Somewhat difficult
- Very difficult

Week	Training	Feasibility LEFT	Feasibility RIGHT
7	1		
	2		
	3		
	4		
	5		
8	1		
	2		
	3		
	4		
	5		
9	1		
	2		
	3		
	4		
	5		
10	1		
	2		
	3		
	4		
	5		
11	1		
	2		
	3		
	4		
	5		
12	1		
	2		
	3		
	4		
	5		

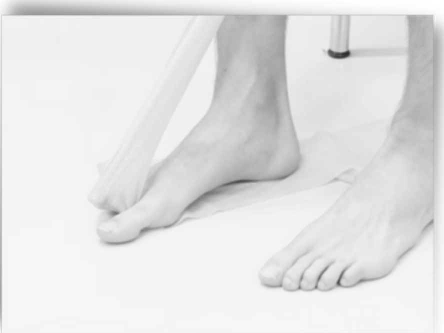

Exercise 9: Lesser toes flexion against resistance

Equipment:

- Resistance band

Instruction:

- Take a seat on a chair and place your feet flat on the floor at hip width;
- Place the resistance band under your foot with one end under the heel and the other around the four smaller toes;
- Stand up with the band in your hand to create tension;
- Push the toes into the band;
- Hold this for **5 seconds**;
- After you have done all the repetitions, repeat everything with your other foot.

The more tension on the band, the harder the exercise

Keep the ball of your foot on the floor

Position	Side	Repetitions	Rest	Repetitions	Moment of adjustment (week, training#)
Seated	L / R	10 x 5 sec			

Very easy
Somewhat easy
Neutral
Somewhat difficult
Very difficult

Exercise 9: Lesser toes flexion against resistance

Week	Training	Feasibility LEFT	Feasibility RIGHT
1	1		
	2		
	3		
	4		
	5		
2	1		
	2		
	3		
	4		
	5		
3	1		
	2		
	3		
	4		
	5		
4	1		
	2		
	3		
	4		
	5		
5	1		
	2		
	3		
	4		
	5		
6	1		
	2		
	3		
	4		
	5		

Very easy
Somewhat easy
Neutral
Somewhat difficult
Very difficult

Exercise 9: Lesser toes flexion against resistance

Week	Training	Feasibility LEFT	Feasibility RIGHT
7	1		
	2		
	3		
	4		
	5		
8	1		
	2		
	3		
	4		
	5		
9	1		
	2		
	3		
	4		
	5		
10	1		
	2		
	3		
	4		
	5		
11	1		
	2		
	3		
	4		
	5		
12	1		
	2		
	3		
	4		
	5		

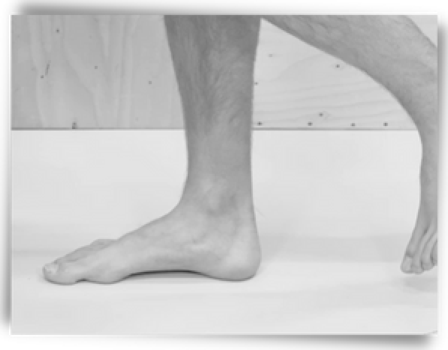

Exercise 10: single leg stance

Instruction:

- When doing this exercise, always hold the back of a chair, or something similar;
- Place your feet flat on the floor at hip width;
- Then lift one foot slightly off the floor;
- Keep the supporting leg bent;
- Try to hold this position according to the duration in the table below;
- Repeat the exercise with your other foot.

Put your foot back on the floor briefly when you lose your balance

Keep your eyes straight ahead

Position	Side	Repetitions	Rest	Repetitions	Moment of adjustment (week, training#)
Single leg stance	L / R	20 sec			

Exercise 10: single leg stance

- Very easy
- Somewhat easy
- Neutral
- Somewhat difficult
- Very difficult

Week	Training	Feasibility LEFT	Feasibility RIGHT
1	1		
	2		
	3		
	4		
	5		
2	1		
	2		
	3		
	4		
	5		
3	1		
	2		
	3		
	4		
	5		
4	1		
	2		
	3		
	4		
	5		
5	1		
	2		
	3		
	4		
	5		
6	1		
	2		
	3		
	4		
	5		

Exercise 10: single leg stance

- Very easy
- Somewhat easy
- Neutral
- Somewhat difficult
- Very difficult

Week	Training	Feasibility LEFT	Feasibility RIGHT
7	1		
	2		
	3		
	4		
	5		
8	1		
	2		
	3		
	4		
	5		
9	1		
	2		
	3		
	4		
	5		
10	1		
	2		
	3		
	4		
	5		
11	1		
	2		
	3		
	4		
	5		
12	1		
	2		
	3		
	4		
	5		

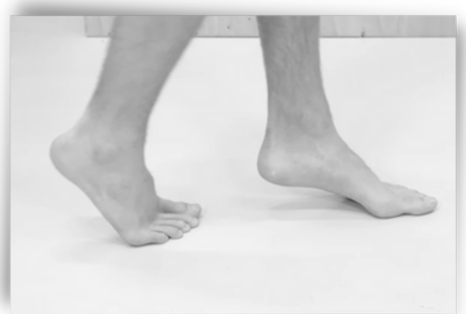

Exercise 11: toe walking

Only when heel raising succeeds properly

Instruction:

- When doing this exercise, always walk past something you can have support from, such as a table or wall;
- Stand on your toes;
- Try to walk on your toes for **20 seconds**.

Try to tiptoe as high as possible

Rest when needed

Position	Side	Repetitions	Rest	Repetitions	Moment of adjustment (week, training#)
n/a	n/a	20 sec			

Exercise 11: toe walking

- Very easy
- Somewhat easy
- Neutral
- Somewhat difficult
- Very difficult

Week	Training	Feasibility LEFT	Feasibility RIGHT
1	1		
	2		
	3		
	4		
	5		
2	1		
	2		
	3		
	4		
	5		
3	1		
	2		
	3		
	4		
	5		
4	1		
	2		
	3		
	4		
	5		
5	1		
	2		
	3		
	4		
	5		
6	1		
	2		
	3		
	4		
	5		

Exercise 11: toe walking

- Very easy
- Somewhat easy
- Neutral
- Somewhat difficult
- Very difficult

Week	Training	Feasibility LEFT	Feasibility RIGHT
7	1		
	2		
	3		
	4		
	5		
8	1		
	2		
	3		
	4		
	5		
9	1		
	2		
	3		
	4		
	5		
10	1		
	2		
	3		
	4		
	5		
11	1		
	2		
	3		
	4		
	5		
12	1		
	2		
	3		
	4		
	5		

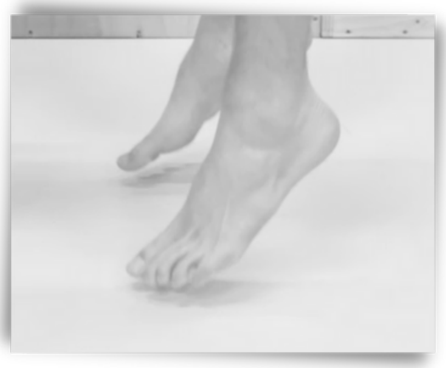

Exercise 12: Hopping

Only when toe walking succeeds properly

Instruction:

- Check the table below to see if you are performing this exercise on two legs or one leg;
- Always hold the back of a chair, or something similar, when doing this exercise;
- Place your feet flat on the floor at hip width;
- Lift the heel(s) slightly off the floor;
- Now make a small jump, with the toes only just off the ground;
- Land on your toes;
- Did you perform this exercise on one leg? Then repeat everything with your other foot.

The toes need only just, if at all, come off the ground

Position	Side	Repetitions	Rest	Repetitions	Moment of adjustment (week, training#)
Double leg stance	L / R	10 x			

Exercise 12: Hopping

- Very easy
- Somewhat easy
- Neutral
- Somewhat difficult
- Very difficult

Week	Training	Feasibility LEFT	Feasibility RIGHT
1	1		
	2		
	3		
	4		
	5		
2	1		
	2		
	3		
	4		
	5		
3	1		
	2		
	3		
	4		
	5		
4	1		
	2		
	3		
	4		
	5		
5	1		
	2		
	3		
	4		
	5		
6	1		
	2		
	3		
	4		
	5		

Exercise 12: Hopping

- Very easy
- Somewhat easy
- Neutral
- Somewhat difficult
- Very difficult

Week	Training	Feasibility LEFT	Feasibility RIGHT
7	1		
	2		
	3		
	4		
	5		
8	1		
	2		
	3		
	4		
	5		
9	1		
	2		
	3		
	4		
	5		
10	1		
	2		
	3		
	4		
	5		
11	1		
	2		
	3		
	4		
	5		
12	1		
	2		
	3		
	4		
	5		

Week 1	
For each training, please describe here in a few words how you experienced the **unsupervised training** and how long it took.	
Training #1	
Training #2	
Training #3	
Training #4	
Have you completed the **supervised training** this week?	Yes / No<br>If yes, please indicate the date:
Indicate here any **discomfort** you experienced **while** doing the exercises.<br>*For example: lower foot cramping when performing exercise x*	
Indicate here what **physical activities** of at least moderate intensity you engaged in outside the foot training that lasted >10 min. consecutively.	
Indicate here any mobility-related **discomforts** you experienced **outside** the foot training, *such as pain in your ankle, foot, leg or lower back*. Please also indicate here if, unexpectedly, you have fallen.	

**Week 2**	
For each training, please describe here in a few words how you experienced the **unsupervised training** and how long it took.	
Training #1	
Training #2	
Training #3	
Training #4	
Have you completed the **supervised training** this week?	Yes / No<br>If yes, please indicate the date:
Indicate here any **discomfort** you experienced **while** doing the exercises.<br>*For example: lower foot cramping when performing exercise x*	
Indicate here what **physical activities** of at least moderate intensity you engaged in outside the foot training that lasted >10 min. consecutively.	
Indicate here any mobility-related **discomforts** you experienced **outside** the foot training, *such as pain in your ankle, foot, leg or lower back*. Please also indicate here if, unexpectedly, you have fallen.	

Week 3	
For each training, please describe here in a few words how you experienced the **unsupervised training** and how long it took.	
Training #1	
Training #2	
Training #3	
Training #4	
Have you completed the **supervised training** this week?	Yes / No<br>If yes, please indicate the date:
Indicate here any **discomfort** you experienced **while** doing the exercises.<br>*For example: lower foot cramping when performing exercise x*	
Indicate here what **physical activities** of at least moderate intensity you engaged in outside the foot training that lasted >10 min. consecutively.	
Indicate here any mobility-related **discomforts** you experienced **outside** the foot training, *such as pain in your ankle, foot, leg or lower back.* Please also indicate here if, unexpectedly, you have fallen.	

**Week 4**	
For each training, please describe here in a few words how you experienced the **unsupervised training** and how long it took.	
Training #1	
Training #2	
Training #3	
Training #4	
Have you completed the **supervised training** this week?	Yes / No<br>If yes, please indicate the date:
Indicate here any **discomfort** you experienced **while** doing the exercises.<br>*For example: lower foot cramping when performing exercise x*	
Indicate here what **physical activities** of at least moderate intensity you engaged in outside the foot training that lasted >10 min. consecutively.	
Indicate here any mobility-related **discomforts** you experienced **outside** the foot training, *such as pain in your ankle, foot, leg or lower back.* Please also indicate here if, unexpectedly, you have fallen.	

Week 5	
For each training, please describe here in a few words how you experienced the **unsupervised training** and how long it took.	
Training #1	
Training #2	
Training #3	
Training #4	
Have you completed the **supervised training** this week?	Yes / No<br>If yes, please indicate the date:
Indicate here any **discomfort** you experienced **while** doing the exercises.<br>*For example: lower foot cramping when performing exercise x*	
Indicate here what **physical activities** of at least moderate intensity you engaged in outside the foot training that lasted >10 min. consecutively.	
Indicate here any mobility-related **discomforts** you experienced **outside** the foot training, *such as pain in your ankle, foot, leg or lower back.* Please also indicate here if, unexpectedly, you have fallen.	

**Week 6**	
For each training, please describe here in a few words how you experienced the **unsupervised training** and how long it took.	
Training #1	
Training #2	
Training #3	
Training #4	
Have you completed the **supervised training** this week?	Yes / No<br>If yes, please indicate the date:
Indicate here any **discomfort** you experienced **while** doing the exercises.<br>*For example: lower foot cramping when performing exercise x*	
Indicate here what **physical activities** of at least moderate intensity you engaged in outside the foot training that lasted >10 min. consecutively.	
Indicate here any mobility-related **discomforts** you experienced **outside** the foot training, *such as pain in your ankle, foot, leg or lower back.* Please also indicate here if, unexpectedly, you have fallen.	

**Week 7**	
For each training, please describe here in a few words how you experienced the **unsupervised training** and how long it took.	
Training #1	
Training #2	
Training #3	
Training #4	
Have you completed the **supervised training** this week?	Yes / No<br>If yes, please indicate the date:
Indicate here any **discomfort** you experienced **while** doing the exercises.<br>*For example: lower foot cramping when performing exercise x*	
Indicate here what **physical activities** of at least moderate intensity you engaged in outside the foot training that lasted >10 min. consecutively.	
Indicate here any mobility-related **discomforts** you experienced **outside** the foot training, *such as pain in your ankle, foot, leg or lower back*. Please also indicate here if, unexpectedly, you have fallen.	

**Week 8**	
For each training, please describe here in a few words how you experienced the **unsupervised training** and how long it took.	
Training #1	
Training #2	
Training #3	
Training #4	
Have you completed the **supervised training** this week?	Yes / No<br>If yes, please indicate the date:
Indicate here any **discomfort** you experienced **while** doing the exercises.<br>*For example: lower foot cramping when performing exercise x*	
Indicate here what **physical activities** of at least moderate intensity you engaged in outside the foot training that lasted >10 min. consecutively.	
Indicate here any mobility-related **discomforts** you experienced **outside** the foot training, *such as pain in your ankle, foot, leg or lower back.* Please also indicate here if, unexpectedly, you have fallen.	

**Week 9**	
For each training, please describe here in a few words how you experienced the **unsupervised training** and how long it took.	
Training #1	
Training #2	
Training #3	
Training #4	
Have you completed the **supervised training** this week?	Yes / No<br>If yes, please indicate the date:
Indicate here any **discomfort** you experienced **while** doing the exercises.<br>*For example: lower foot cramping when performing exercise x*	
Indicate here what **physical activities** of at least moderate intensity you engaged in outside the foot training that lasted >10 min. consecutively.	
Indicate here any mobility-related **discomforts** you experienced **outside** the foot training, *such as pain in your ankle, foot, leg or lower back*. Please also indicate here if, unexpectedly, you have fallen.	

Week 10	
For each training, please describe here in a few words how you experienced the **unsupervised training** and how long it took.	
Training #1	
Training #2	
Training #3	
Training #4	
Have you completed the **supervised training** this week?	Yes / No<br>If yes, please indicate the date:
Indicate here any **discomfort** you experienced **while** doing the exercises.<br>*For example: lower foot cramping when performing exercise x*	
Indicate here what **physical activities** of at least moderate intensity you engaged in outside the foot training that lasted >10 min. consecutively.	
Indicate here any mobility-related **discomforts** you experienced **outside** the foot training, *such as pain in your ankle, foot, leg or lower back*. Please also indicate here if, unexpectedly, you have fallen.	

**Week 11**	
For each training, please describe here in a few words how you experienced the **unsupervised training** and how long it took.	
Training #1	
Training #2	
Training #3	
Training #4	
Have you completed the **supervised training** this week?	Yes / No<br>If yes, please indicate the date:
Indicate here any **discomfort** you experienced **while** doing the exercises.<br>*For example: lower foot cramping when performing exercise x*	
Indicate here what **physical activities** of at least moderate intensity you engaged in outside the foot training that lasted >10 min. consecutively.	
Indicate here any mobility-related **discomforts** you experienced **outside** the foot training, *such as pain in your ankle, foot, leg or lower back.* Please also indicate here if, unexpectedly, you have fallen.	

**Week 12**	
For each training, please describe here in a few words how you experienced the **unsupervised training** and how long it took.	
Training #1	
Training #2	
Training #3	
Training #4	
Have you completed the **supervised training** this week?	Yes / No<br>If yes, please indicate the date:
Indicate here any **discomfort** you experienced **while** doing the exercises.<br>*For example: lower foot cramping when performing exercise x*	
Indicate here what **physical activities** of at least moderate intensity you engaged in outside the foot training that lasted >10 min. consecutively.	
Indicate here any mobility-related **discomforts** you experienced **outside** the foot training, *such as pain in your ankle, foot, leg or lower back.* Please also indicate here if, unexpectedly, you have fallen.	
