# Supplementary material for: Effects of a 12-week intrinsic foot muscle strengthening training (STIFF) on gait in older adults: a parallel randomized controlled trial protocol
Source: BMC Sports Sci Med Rehabil. 2024 Jul 20;16:158. doi: 10.1186/s13102-024-00944-z (PMC11542310; doi:10.1186/s13102-024-00944-z)
Supplement: Supplementary file 4 — Additional file 4. Trainer’s guide. [file 13102_2024_944_MOESM4_ESM.pdf]

TRAINER:

STIFF TRAINER'S GUIDE

For the study into foot muscles and the ability to move in older adults

Fontys Allied Health Professions
in collaboration with
SGE

Your contribution to the study

You are one of the trainers of the study into the effect of foot training on balance and walking in older adults. All the information about this study can be found in the protocol, the participant information letter, the training guide for the participants in the intervention group and the diary of the control group. You will also complete the train the trainer workshop. It is important that you have thoroughly familiarized yourself with the information. This trainer's guide contains some additional information and tools that you will use to properly guide and monitor the participants.

As a trainer, you have a very important job in this study. From the moment the research support person has informed the chief trainer that a participant has been assigned to either the intervention group or the control group, your job is to:

1. introduce the control or intervention guidelines to the participant
2. make the weekly arrangements with the participant for the training sessions with trainer (intervention) or weekly call the participants (control)
3. organize the group training sessions on campus
4. instruct and motivate the participant on the exercises
5. encourage the participant to adhere to the program
6. determine and document the progression level of the exercises
7. report any discomfort during or outside the training that relates to mobility to the research support person and consult with the researcher (without disclosing the group assignment)

It is very important for the study that the researcher, Lydia Willemse, does not know which group the participants are assigned to. Therefore, emphasize this during the final training session or call.

Do not use the participant's name in any documentation, but instead use the participant ID

Overview of all participants

This overview lists all the participants, the group assignment and the trainer who will take care of the participant during the 12 weeks.

ID	Group (I / C)	Trainer	Location (home / campus)	Trainingsgroup
99	I	PP	home	Tuesday 14.00-14.30 / n/a

Overview participants per trainer

This overview lists each trainer's participants, the appointments made and a checkbox for if a participant actually did the supervised training (intervention group) or if the trainer discussed the diary with the participant (control group).

**ID**	14					
**Group (I / C)**	I					
**Location (home/campus)**	home					
**#1**	2-10, 10.00 ☐	☐	☐	☐	☐	☐
**#2**	☐	☐	☐	☐	☐	☐
**#3**	☐	☐	☐	☐	☐	☐
**#4**	☐	☐	☐	☐	☐	☐
**#5**	☐	☐	☐	☐	☐	☐
**#6**	☐	☐	☐	☐	☐	☐
**#7**	☐	☐	☐	☐	☐	☐
**#8**	☐	☐	☐	☐	☐	☐
**#9**	☐	☐	☐	☐	☐	☐
**#10**	☐	☐	☐	☐	☐	☐
**#11**	☐	☐	☐	☐	☐	☐
**#12**	☐	☐	☐	☐	☐	☐

The supervised training

Each training session, keep a fixed order of the following steps so you can't forget anything.

1. Explain what you and the participant will do during training
2. Using the diary, ask if any discomforts occurred during or outside the training that are related to mobility. If yes, see flow chart.
3. Using the diary, ask how the unsupervised training sessions went
4. **Go through each exercise from the training guide together,** score the motor performance, note the exercise level in the participant's training guide and mark the completed training
   - Watch instructional video together
   - Verbally explaining the exercise
   - Perform the exercise together
   - Have the participant circle the smiley himself/herself in the training guide
5. Make an appointment for the next supervised training session
6. Emphasize the importance of reporting mobility discomforts during or outside the training to the trainer
7. Motivate the participant to do the exercises according to the program and using the videos

! If a participant wants to quit the study: notify the research support person immediately

Progression in the level of exercises

In **week 1**, always start with the **lowest level**. When advancing to the next level?
The participant perceives the exercise as very easy **AND** maximum motor performance during all repetitions
Write down the adjusted instructions for the next level on the left page of the training book

Exercise	Repetitions	Posture	Level +	Level ++	Level +++
1: Big toe and lesser toes extension	10 x 5 sec	double leg stance	30 sec rest + 5 x 5 sec	not 5 x but 10 x	30 sec rest + 5 x 5 sec
2: Toe spread and squeeze	10 x 5 sec	seated	30 sec rest + 5 x 5 sec	not 5 x but 10 x	30 sec rest + 5 x 5 sec
3: Short foot exercise	5 x 3 sec	double leg stance	single leg stance	30 sec rest + 5 x 3 sec	30 sec rest + 5 x 3 sec
4: towel curl	5 x 2 sec	seated	30 sec rest + 5 x 2 sec	idem	idem
5: Marble pick up	5 x 2 sec	seated	30 sec rest + 5 x 2 sec	idem	idem
6: Big toe and lesser toes press	10 x 5 sec	double leg stance	30 sec rest + 5 x 5sec	not 5 x but 10 x	30 sec rest + 5 x 5 sec
7: Heel raise	10 x 2 sec	seated	double leg stance	single leg stance	30 sec rest + 5 x 2 sec
8: Big toe flexion against resistance	10 x 5 sec	zittend, yellow band	red band	green band	
9: Lesser toes flexion against resistance	10 x 5 sec	seated, yellow band	red band	green band	
10: Single leg stance	20 sec	single leg stance	+ 10 sec	idem	idem
11: Toe walking	1 x 20 sec	double leg stance	30 sec rest + 1 x 20 sec	idem	idem
12: Hopping	10 x	both legs	one leg	30 sec rest + 5 x	not 5 x, but 10 x

Only when heel raising level +

Only when toe walking ' level +

What if the training takes >30 minutes?

The training time may be shortened if the participant spends more than 30 minutes training. At least 20 minutes of actual training must be done, excluding instructions, chitchatting and rest between exercises. A participant may train for longer than 30 minutes at their own discretion. Follow these instructions to shorten the training sessions:

1. Training with two feet at the same time (only if the quality of movement is not diminished by this).
2. Use the long video to go through all the exercises in one run.
3. Discuss with the participant which exercises will be shortened or omitted.
   - In the first weeks: only shorten exercises by repetitions, preferably do not omit exercises.
   - In the following weeks: omit exercises or combine fewer reps and omitting exercises.

When exercises need to be omitted, adhere to the guideline below so that all muscles are addressed in the training program. Note the adjustments in the training guide in the table below the explanation of the exercise and the reason why it was adjusted.

**Exercises that must be kept in the training**	**At least one of the following exercises must be kept in the training**	**May be omitted from the training**
• Grote tenen en kleine tenen in de grond duwen<br>• Tenen spreiden en samenknijpen<br>• Short foot exercise	• Handdoek grijpen / knikker grijpen / grote teen en kleine teen flexie met weerstand<br>• Tenenstand / tenen lopen / hoppen	Big toe and lesser toe extension

Flow chart discomfort

In the following way, discomforts are reported and followed up. These are not only discomforts that can be directly linked to foot training. In general, it is about discomforts related to mobility that occur during or outside the training. For example, pain in the lower back.

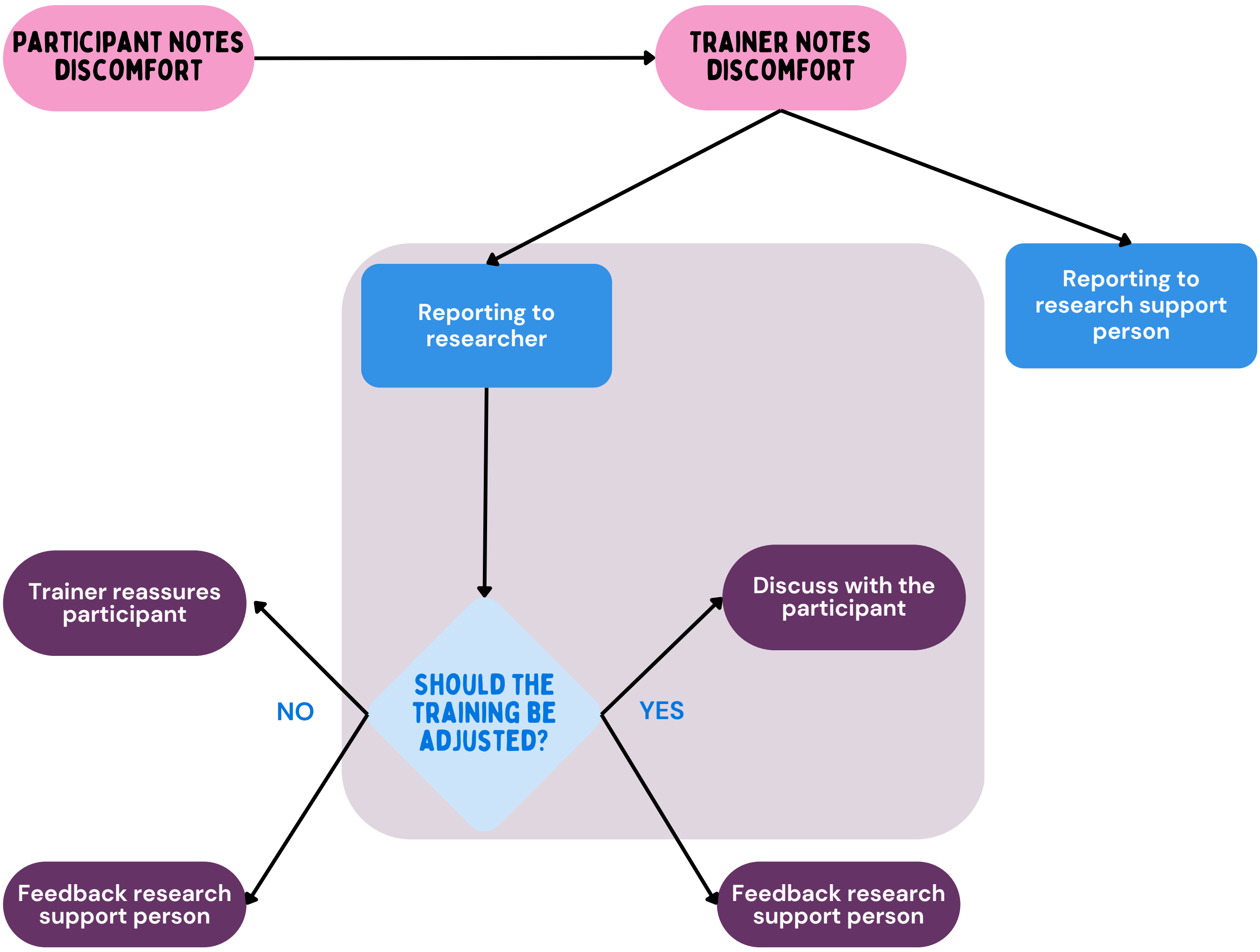

Motor performance

ID:

Motor performance:

0: no movement or position cannot be maintained
1: exercise can be partially completed or with difficulty or compensation
2: exercise can be completed with typical performance

Exercise	Side	Wk1	Wk2	Wk3	Wk4	Wk5	Wk6	Wk7	Wk8	Wk9	Wk10	Wk11	Wk12
1	L												
	R												
2	L												
	R												
3	L												
	R												
4	L												
	R												
5	L												
	R												
6	L												
	R												
7	L												
	R												
8	L												
	R												
9	L												
	R												
10	L												
	R												
11	L												
	R												
12	L												
	R												

Motor performance

ID:

Motor performance:
0: no movement or position cannot be maintained
1: exercise can be partially completed or with difficulty or compensation
2: exercise can be completed with typical performance

Exercise	Side	Wk1	Wk2	Wk3	Wk4	Wk5	Wk6	Wk7	Wk8	Wk9	Wk10	Wk11	Wk12
1	L												
	R												
2	L												
	R												
3	L												
	R												
4	L												
	R												
5	L												
	R												
6	L												
	R												
7	L												
	R												
8	L												
	R												
9	L												
	R												
10	L												
	R												
11	L												
	R												
12	L												
	R												

Motor performance

ID:

Motor performance:
0: no movement or position cannot be maintained
1: exercise can be partially completed or with difficulty or compensation
2: exercise can be completed with typical performance

Exercise	Side	Wk1	Wk2	Wk3	Wk4	Wk5	Wk6	Wk7	Wk8	Wk9	Wk10	Wk11	Wk12
1	L												
	R												
2	L												
	R												
3	L												
	R												
4	L												
	R												
5	L												
	R												
6	L												
	R												
7	L												
	R												
8	L												
	R												
9	L												
	R												
10	L												
	R												
11	L												
	R												
12	L												
	R												

Motor performance

ID:

Motor performance:

0: no movement or position cannot be maintained
1: exercise can be partially completed or with difficulty or compensation
2: exercise can be completed with typical performance

Exercise	Side	Wk1	Wk2	Wk3	Wk4	Wk5	Wk6	Wk7	Wk8	Wk9	Wk10	Wk11	Wk12
1	L												
	R												
2	L												
	R												
3	L												
	R												
4	L												
	R												
5	L												
	R												
6	L												
	R												
7	L												
	R												
8	L												
	R												
9	L												
	R												
10	L												
	R												
11	L												
	R												
12	L												
	R												
