# Supplementary material for: Effects of a 12-week intrinsic foot muscle strengthening training (STIFF) on gait in older adults: a parallel randomized controlled trial protocol
Source: BMC Sports Sci Med Rehabil. 2024 Jul 20;16:158. doi: 10.1186/s13102-024-00944-z (PMC11542310; doi:10.1186/s13102-024-00944-z)
Supplement: Supplementary file 5 — Additional file 5. Diary (control). [file 13102_2024_944_MOESM5_ESM.pdf]

ID:

STIFF DIARY

For the study into foot muscles and the ability to move in older adults

Fontys Allied Health Professions
in collaboration with
SGE

Fontys STIFF SGE

Your participation in the study

You are participating in the study on the effect of foot training on balance and walking. For this, you are assigned to the group that only keeps a diary and completes the measurements. You can read more about this on the following pages.

It is of utmost importance for the study that the researcher, Lydia Willemse, does not know that you are assigned to the control group. Therefore, please try to keep this unrevealed when you see her at the last measurement.

The timeline of the study

	MEASUREMENT 1A: HOME	Date measurement 1A:
	MEASUREMENT 1B: FONTYS	Date measurement 1B:
4 x self training<br>1 x with trainer	WEEK 1	Date with trainer:<br>Location:
4 x self training<br>1 x with trainer	WEEK 2	Date with trainer:<br>Location:
4 x self training<br>1 x with trainer	WEEK 3	Date with trainer:<br>Location:
4 x self training<br>1 x with trainer	WEEK 4	Date with trainer:<br>Location:
4 x self training<br>1 x with trainer	WEEK 5	Date with trainer:<br>Location:
4 x self training<br>1 x with trainer	WEEK 6	Date with trainer:<br>Location:
4 x self training<br>1 x with trainer	WEEK 7	Date with trainer:<br>Location:
4 x self training<br>1 x with trainer	WEEK 8	Date with trainer:<br>Location:
4 x self training<br>1 x with trainer	WEEK 9	Date with trainer:<br>Location:
4 x self training<br>1 x with trainer	WEEK 10	Date with trainer:<br>Location:
4 x self training<br>1 x with trainer	WEEK 11	Date with trainer:<br>Location:
4 x self training<br>1 x with trainer	WEEK 12	Date with trainer:<br>Location:
	MEASUREMENT 2: FONTYS	

Location Fontys:
Rachelsmolen 1
Building R12
5612 MA Eindhoven

The diary

For the study, it is important that you keep a diary, in which you write down your physcial activities and, if any, discomfort you encountered. This diary can be found on the following pages. One of the trainers below will call you weekly to go through what you filled out there.

Please write down only those activities that you perceive to be at least moderately intense and that last at least in bouts of 10 minutes. For example, going for a walk or riding a bike. You do not need to write down household chores.

**Week 1**	
Indicate here what **physical activities** of at least moderate intensity you engaged in that lasted >10 min. consecutively.	
Monday	
Tuesday	
Wednesday	
Thursday	
Friday	
Saturday	
Sunday	
Indicate here any **mobility-related discomforts** you experienced, *such as pain in your ankle, foot, leg or lower back.* Please also indicate here if, unexpectedly, you have fallen.	

**Week 2**	
Indicate here what **physical activities** of at least moderate intensity you engaged in that lasted >10 min. consecutively.	
Monday	
Tuesday	
Wednesday	
Thursday	
Friday	
Saturday	
Sunday	
Indicate here any **mobility-related discomforts** you experienced, *such as pain in your ankle, foot, leg or lower back.* Please also indicate here if, unexpectedly, you have fallen.	

Week 3	
Indicate here what **physical activities** of at least moderate intensity you engaged in that lasted >10 min. consecutively.	
Monday	
Tuesday	
Wednesday	
Thursday	
Friday	
Saturday	
Sunday	
Indicate here any **mobility-related discomforts** you experienced, *such as pain in your ankle, foot, leg or lower back.* Please also indicate here if, unexpectedly, you have fallen.	

**Week 4**	
Indicate here what **physical activities** of at least moderate intensity you engaged in that lasted >10 min. consecutively.	
Monday	
Tuesday	
Wednesday	
Thursday	
Friday	
Saturday	
Sunday	
Indicate here any **mobility-related discomforts** you experienced, *such as pain in your ankle, foot, leg or lower back.* Please also indicate here if, unexpectedly, you have fallen.	

**Week 5**	
Indicate here what **physical activities** of at least moderate intensity you engaged in that lasted >10 min. consecutively.	
Monday	
Tuesday	
Wednesday	
Thursday	
Friday	
Saturday	
Sunday	
Indicate here any **mobility-related discomforts** you experienced, *such as pain in your ankle, foot, leg or lower back.* Please also indicate here if, unexpectedly, you have fallen.	

**Week 6**	
Indicate here what **physical activities** of at least moderate intensity you engaged in that lasted >10 min. consecutively.	
Monday	
Tuesday	
Wednesday	
Thursday	
Friday	
Saturday	
Sunday	
Indicate here any **mobility-related discomforts** you experienced, *such as pain in your ankle, foot, leg or lower back.* Please also indicate here if, unexpectedly, you have fallen.	

**Week 7**	
Indicate here what **physical activities** of at least moderate intensity you engaged in that lasted >10 min. consecutively.	
Monday	
Tuesday	
Wednesday	
Thursday	
Friday	
Saturday	
Sunday	
Indicate here any **mobility-related discomforts** you experienced, *such as pain in your ankle, foot, leg or lower back.* Please also indicate here if, unexpectedly, you have fallen.	

Week 8	
Indicate here what **physical activities** of at least moderate intensity you engaged in that lasted >10 min. consecutively.	
Monday	
Tuesday	
Wednesday	
Thursday	
Friday	
Saturday	
Sunday	
Indicate here any **mobility-related discomforts** you experienced, *such as pain in your ankle, foot, leg or lower back.* Please also indicate here if, unexpectedly, you have fallen.	

Week 9	
Indicate here what **physical activities** of at least moderate intensity you engaged in that lasted >10 min. consecutively.	
Monday	
Tuesday	
Wednesday	
Thursday	
Friday	
Saturday	
Sunday	
Indicate here any **mobility-related discomforts** you experienced, *such as pain in your ankle, foot, leg or lower back.* Please also indicate here if, unexpectedly, you have fallen.	

**Week 10**	
Indicate here what **physical activities** of at least moderate intensity you engaged in that lasted >10 min. consecutively.	
Monday	
Tuesday	
Wednesday	
Thursday	
Friday	
Saturday	
Sunday	
Indicate here any **mobility-related discomforts** you experienced, *such as pain in your ankle, foot, leg or lower back.* Please also indicate here if, unexpectedly, you have fallen.	

**Week 11**	
Indicate here what **physical activities** of at least moderate intensity you engaged in that lasted >10 min. consecutively.	
Monday	
Tuesday	
Wednesday	
Thursday	
Friday	
Saturday	
Sunday	
Indicate here any **mobility-related discomforts** you experienced, *such as pain in your ankle, foot, leg or lower back.* Please also indicate here if, unexpectedly, you have fallen.	

Week 12	
Indicate here what **physical activities** of at least moderate intensity you engaged in that lasted >10 min. consecutively.	
Monday	
Tuesday	
Wednesday	
Thursday	
Friday	
Saturday	
Sunday	
Indicate here any **mobility-related discomforts** you experienced, *such as pain in your ankle, foot, leg or lower back.* Please also indicate here if, unexpectedly, you have fallen.	
